# A TLR4/TRAF6-dependent signaling pathway mediates NCoR coactivator complex formation for inflammatory gene activation

**DOI:** 10.1073/pnas.2316104121

**Published:** 2024-01-02

**Authors:** Yohei Abe, Eric R. Kofman, Zhengyu Ouyang, Grisel Cruz-Becerra, Nathanael J. Spann, Jason S. Seidman, Ty D. Troutman, Joshua D. Stender, Havilah Taylor, Weiwei Fan, Verena M. Link, Zeyang Shen, Juro Sakai, Michael Downes, Ronald M. Evans, James T. Kadonaga, Michael G. Rosenfeld, Christopher K. Glass

**Affiliations:** ^a^Department of Cellular and Molecular Medicine, University of California San Diego, La Jolla, CA 92093; ^b^Stem Cell Program, University of California San Diego, La Jolla, CA 92093; ^c^Institute for Genomic Medicine, University of California San Diego, La Jolla, CA 92093; ^d^Department of Molecular Biology, University of California San Diego, La Jolla, CA 92093; ^e^Department of Medicine, University of California San Diego, La Jolla, CA 92093; ^f^Division of Allergy and Immunology, Cincinnati Children's Hospital Medical Center, Department of Pediatrics, University of Cincinnati, Cincinnati, OH 45229; ^g^Department and School of Medicine, University of California San Diego, La Jolla, CA 92093; ^h^Gene Expression Laboratory, Salk Institute for Biological Studies, La Jolla, CA 92037; ^i^Faculty of Biology, Department II, Ludwig-Maximilians Universität München, Munich 82152, Germany; ^j^Department of Bioengineering, Jacobs School of Engineering, University of California San Diego, La Jolla, CA 92093; ^k^Division of Metabolic Medicine, Research Center for Advanced Science and Technology, The University of Tokyo, Tokyo 153-8904, Japan; ^l^Division of Molecular Physiology and Metabolism, Tohoku University Graduate School of Medicine, Sendai 980-8575, Japan

**Keywords:** macrophage, TRAF6, TLR4, NCoR, PGC1β

## Abstract

The nuclear receptor corepressor (NCoR) forms a complex with histone deacetylase 3 (HDAC3) that mediates repressive functions of unliganded nuclear receptors and other transcriptional repressors by deacetylation of histone substrates. Recent studies provide evidence that NCoR/HDAC3 complexes can also exert coactivator functions in brown adipocytes by deacetylating and activating PPARγ coactivator 1α (PGC1α) and that signaling via receptor activator of nuclear factor kappa-B (RANK) promotes the formation of a stable NCoR/HDAC3/PGC1β complex that coactivates nuclear factor kappa-B (NFκB)- and activator protein 1 (AP-1)-dependent genes required for osteoclast differentiation. Here, we demonstrate that activation of Toll-like receptor (TLR) 4, but not TLR3, the interleukin 4 (IL4) receptor nor the Type I interferon receptor, also promotes assembly of an NCoR/HDAC3/PGC1β coactivator complex. Receptor-specific utilization of TNF receptor-associated factor 6 (TRAF6) and downstream activation of extracellular signal-regulated kinase 1 (ERK1) and TANK-binding kinase 1 (TBK1) accounts for the common ability of RANK and TLR4 to drive assembly of an NCoR/HDAC3/PGC1β complex in macrophages. ERK1, the p65 component of NFκB, and the p300 histone acetyltransferase (HAT) are also components of the induced complex and are associated with local histone acetylation and transcriptional activation of TLR4-dependent enhancers and promoters. These observations identify a TLR4/TRAF6-dependent signaling pathway that converts NCoR from a corepressor of nuclear receptors to a coactivator of NFκB and AP-1 that may be relevant to functions of NCoR in other developmental and homeostatic processes.

## Significance

The nuclear receptor corepressor NCoR is generally considered to mediate transcriptional repression dependent on its interaction with the histone deacetylase HDAC3. Here, we show that NCoR can be converted from a corepressor to a coactivator of NFκB in macrophages by specific signals that promote the interaction of NCoR and HDAC3 with PGC1β. These findings raise the possibility that many of the functions of NCoR in development, homeostasis and immunity are due to it serving as a coactivator, rather than as a corepressor. Because deacetylation of PBC1β by HDAC3 is required for this switch, these findings also suggest an additional mechanism by which HDAC3 inhibitors exert their biological effects.

Nuclear receptor corepressor (NCoR) and silencing mediator of retinoid and thyroid hormone receptor (SMRT, also known as NCoR2) are broadly expressed transcriptional corepressors that play essential roles in diverse aspects of development and homeostasis (1). The corepressor functions of NCoR and SMRT are dependent on interaction with histone deacetylase 3 (HDAC3), which mainly deacetylates K27 on the histone H3 N-terminal tail (H3K27) (2–4). NCoR and SMRT were initially identified based on their interactions with a subset of unliganded nuclear receptors, including thyroid hormone receptors, retinoic acid receptors, and liver X receptors (LXRs) (5–7). The interaction of NCoR or SMRT with unliganded nuclear receptors bound to their cognate recognition elements at enhancers and promoters results in the recruitment of HDAC3, local histone deacetylation, and transcriptional repression. The binding of activating ligands to each of these receptors causes NCoR/HDAC3 and SMRT/HDAC3 corepressor complexes to dissociate in exchange for coactivator complexes with histone acetyltransferase activity that promote transcriptional activation (8). This paradigm of corepressor/coactivator exchange for signal-dependent gene activation was subsequently extended to other classes of transcription factors, including Bcl6 (9), recombination signal binding protein for immunoglobulin Kappa J region (Rbpj) (10), and members of the activator protein 1 (AP-1) family (11). As a consequence, the phenotypes resulting from cell-specific loss of function of NCoR or SMRT have generally been attributed to loss of corepressor activity.

Unexpectedly, targeted loss of function of NCoR in macrophages resulted in protection from diet-induced insulin resistance and an attenuated, rather than exaggerated, transcriptional response to the Toll-like receptor 4 (TLR4) agonist, lipopolysaccharide (LPS) (12). TLR4 signaling induces strong transcriptional responses in macrophages by activating members of the nuclear factor kappa-B (NFκB), AP-1, and interferon regulatory factor (IRF) families of transcription factors (8). This unexpected phenotype could be partially explained by conventional derepression of LXR target genes encoding fatty acid elongases that catalyze the synthesis of anti-inflammatory omega 3 fatty acids. However, omega 3 fatty acids only partially suppressed LPS-induced genes that were hyporesponsive in NCoR-deficient macrophages (12), which posed the question of why the remaining genes exhibited attenuated responses in NCoR knockout cells. Similarly, loss of HDAC3 resulted in the decreased expression of multiple inflammatory genes in the presence of TLR4 signaling (13). This could be partially explained by the ability of HDAC3 to function as a coactivator for TLR4-dependent genes independent of its catalytic activity (14). However, selective inhibition of HDAC3 enzymatic activity has been shown to reduce inflammatory gene expression in peripheral blood mononuclear cells from rheumatoid arthritis patients (15) and LPS-stimulated macrophages (16). As HDAC3 is only enzymatically active when it interacts with NCoR or SMRT (3, 17, 18), the mechanisms by which HDAC3-specific inhibitors reduce responses to LPS remain enigmatic.

We recently found that in addition to causing insulin sensitization and impaired responses to LPS (12), deletion of NCoR from the myeloid lineage results in a high bone mass phenotype due to defective osteoclast differentiation in response to receptor activator of nuclear factor kappa-B (RANK) signaling (19). By integrating the genome-wide locations of NCoR and HDAC3 with RANK-dependent activation of enhancers and promoters, we found that NCoR/HDAC3 complexes are paradoxically associated with histone acetylation and gene activation rather than histone deacetylation and gene repression. An important clue to explain these findings was the previous discovery that NCoR/HDAC3 deacetylates PPARγ coactivator 1α (PGC1α) as a prerequisite for coactivation of estrogen-related receptor α (ERRα) and induction of thermogenesis in brown adipose tissue. Although PGC1α is not expressed in macrophages, PGC1β is expressed and is further induced by RANK signaling during in vitro osteoclast differentiation. Biochemical studies demonstrated that RANK signaling promotes the assembly of a stable ~2 MDa NCoR/HDAC3/PGC1β complex dependent on noncoding RNAs that functions as a coactivator of RANK-dependent genes (19). We further demonstrated that the formation of this complex alters the specificity of HDAC3, preventing deacetylation of histone H3K27ac in favor of deacetylation and activation of PGC1β, thereby resolving the paradox of why recruitment of NCoR/HDAC3/PGC1β complexes to enhancers and promoters results in histone acetylation, rather than histone deacetylation. TLR4 signaling also induced formation of a NCoR/HDAC3/PGC1β complex with altered substrate specificity of HDAC3 (19). Remarkably, although we observed evidence for conventional corepressor activity of NCoR at nuclear receptor target genes, the major role of NCoR during osteoclast differentiation is as a RANK-induced transcriptional coactivator.

Collectively, these findings force a reevaluation of NCoR complexes in development and homeostasis with respect to their relative functions as corepressors or signal-dependent coactivators. Here, we show that the dominant function of NCoR in the context of TLR4 activation is as a signal-dependent coactivator of NFκB and AP-1 target genes. We present evidence that these observations can be at least partially explained by the common utilization of TNF receptor-associated factor 6 (TRAF6) by TLR4 or RANK in comparison to the other receptor systems studied, which acts through parallel kinase cascades involving extracellular signal-regulated kinase 1 (ERK1) and TANK-binding kinase 1 (TBK1) to convert the NCoR/HDAC3 corepressor complex to a signal-specific NCoR/HDAC3/PGC1β coactivator complex required for inflammatory or osteoclast gene activation, respectively.

## Results

### NCoR-Dependent Response of Macrophages to Extracellular Signals.

**Fig. 1.**
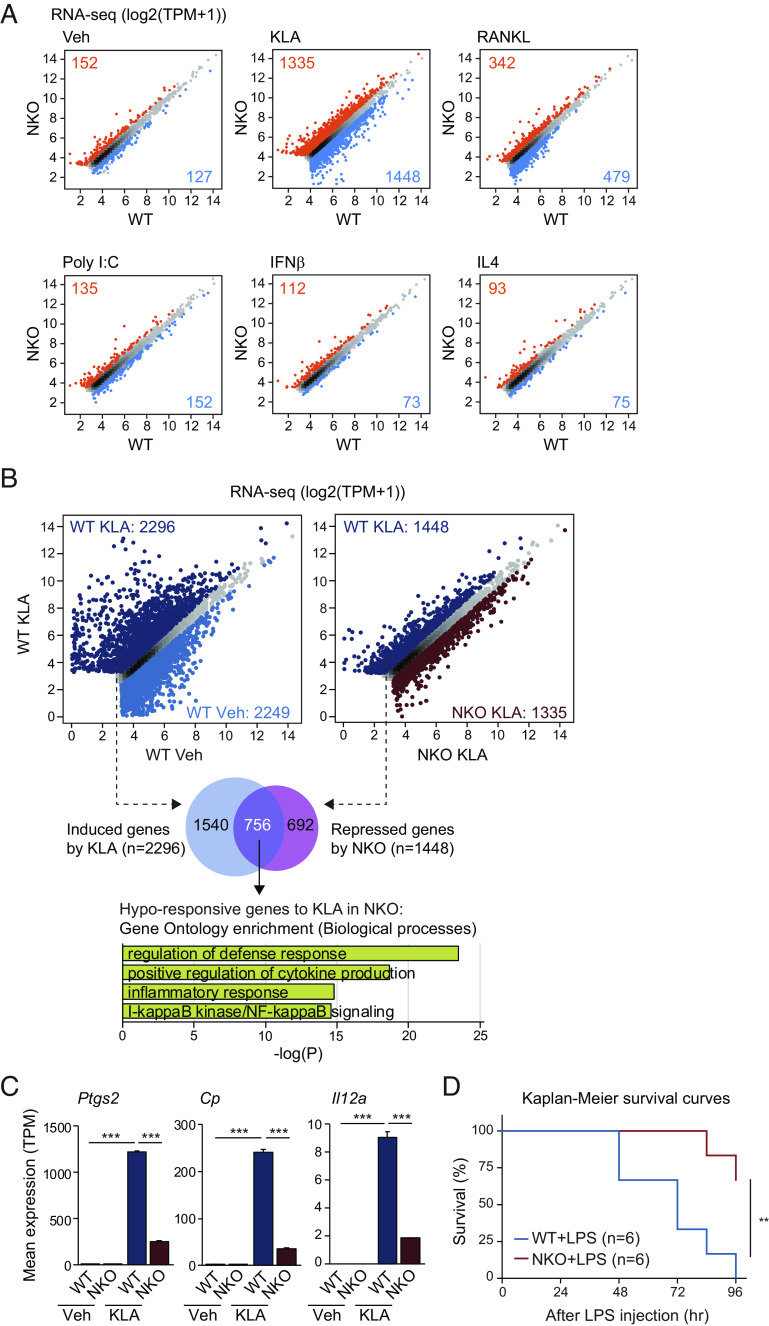
NCoR-dependent response of macrophages to extracellular signals. (*A*) Scatter plots of RNA-seq data showing gene expression in bone marrow–derived macrophages (BMDMs) from wild-type (WT) and NCoR KO (NKO) mice in the presence or absence of KLA, RANKL, Poly I:C, IFNβ, or IL4 (blue dots: significantly NKO-suppressed genes, orange dots: significantly NKO-induced genes, FDR < 0.05, FC > 1.5). (*B*) Scatter plots of RNA-seq data showing KLA-regulated gene expression (*Left*) and NKO-regulated gene expression in the presence of KLA (*Right*) (light blue dots in *Left* panel: significantly KLA-suppressed genes, dark blue dots in *Left* panel: significantly KLA-induced genes, dark red dots in *Right* panel: significantly NKO-induced genes, dark blue dots in *Right* panel: significantly NKO-suppressed genes, FDR < 0.05, FC > 1.5). The overlap between KLA-induced genes (n = 2,296) and NKO-suppressed genes in the presence of KLA (n = 1,448) is shown by the Venn diagram. The significant gene ontology terms associated with the hyporesponsive genes are shown. (*C*) Bar plots for expression of *Ptgs2*, *Cp,* and *Il12a* in WT and NKO BMDMs treated with or without KLA. The significance symbols indicate statistical significance, ***P-adj < 0.001 reported by DESeq2 using the Benjamini–Hochberg method for the multiple-testing correction. (*D*) Kaplan–Meier survival curves of WT and NKO mice subjected to 6 mg/kg LPS by intraperitoneal injection. **P < 0.01 calculated using a Mantel–Cox test. Please see also *SI Appendix*, Fig. S1.

To explore contexts in which NCoR is required for signal-dependent gene activation in macrophages, we crossed *LysM-Cre* mice to *Ncor^f/f^* mice to establish myeloid-specific NCoR-deficient (NKO) mice (12) from which bone marrow-derived macrophages (BMDMs) were differentiated. We then compared the responses of wild type (WT) or NKO BMDMs to KLA (Kdo2 lipid A, an agonist of TLR4 that mimics bacterial lipopolysaccharide) (20), RANKL (an essential factor for osteoclast differentiation) (21–23), poly I:C (a synthetic analog of double stranded RNA that activates TLR3) (24), IFNβ (a major inducer of antiviral activity) (25) and IL4 (a regulator of homeostatic and wound repair phenotypes) (26) (Fig. 1*A*). Under vehicle conditions, NCoR deficiency resulted in derepression of ~150 genes (±>1.5-fold, FDR < 0.05) (Fig. 1*A*), including LXR target genes such as *Abca1* (encoding ATP-binding cassette subfamily A member 1) and *Scd2* (encoding stearoyl-CoA desaturase 2) (*SI Appendix*, Fig. S1*A*), as well as downregulation of ~130 genes (±>1.5-fold, FDR < 0.05) (Fig. 1*A*). In contrast, following KLA stimulation for 6 h (*SI Appendix*, Fig. S1*B*), >1,300 mRNAs were more highly expressed in NCoR-deficient BMDMs, whereas >1,400 mRNAs were expressed at lower levels (Fig. 1*B*). Of the 2,296 mRNAs induced >1.5-fold by KLA (FDR < 0.05) in WT cells, 756 exhibited significantly attenuated responses in NKO cells (Fig. 1*B*), exemplified by *Ptgs2* (encoding cyclooxygenase 2), *Cp* (encoding ceruloplasmin) and *Il12a* (encoding interleukin 12A) (Fig. 1*C*). The 756 genes exhibiting reduced responsiveness to KLA in NKO BMDMs were significantly enriched for functional annotations related to regulation of defense response, positive regulation of cytokine production and NFκB signaling (Fig. 1*B*), in agreement with prior studies (12). Indeed, myeloid deficiency for NCoR significantly protected mice from endotoxin lethality compared to the control mice (Fig. 1*D*), which is correlated with the gene expression profile in NKO macrophages showing hyporesponsiveness to KLA treatment (Fig. 1 *B* and *C*). NCoR deficiency also had a substantial impact on RANKL-dependent gene expression (Fig. 1*A* and *SI Appendix*, Fig. S1*C*), consistent with the previously reported role of NCoR in osteoclastogenesis (19). Surprisingly, there was relatively little impact of NCoR deficiency in the context of TLR3 activation by poly I:C, despite activation of a comparable number of genes to that observed following activation of TLR4 (Fig. 1*A* and *SI Appendix*, Fig. S1*D*). Similarly, responses to IFNβ and IL4 were only modestly affected by NCoR deficiency (Fig. 1*A* and *SI Appendix*, Fig. S1 *E* and *F*). Therefore, the impact of NCoR deficiency on macrophage gene expression is highly dependent on signaling context.

### TLR4 Signaling Induces Recruitment of NCoR/HDAC3 Complex to Enhancers and Promoters.

**Fig. 2.**
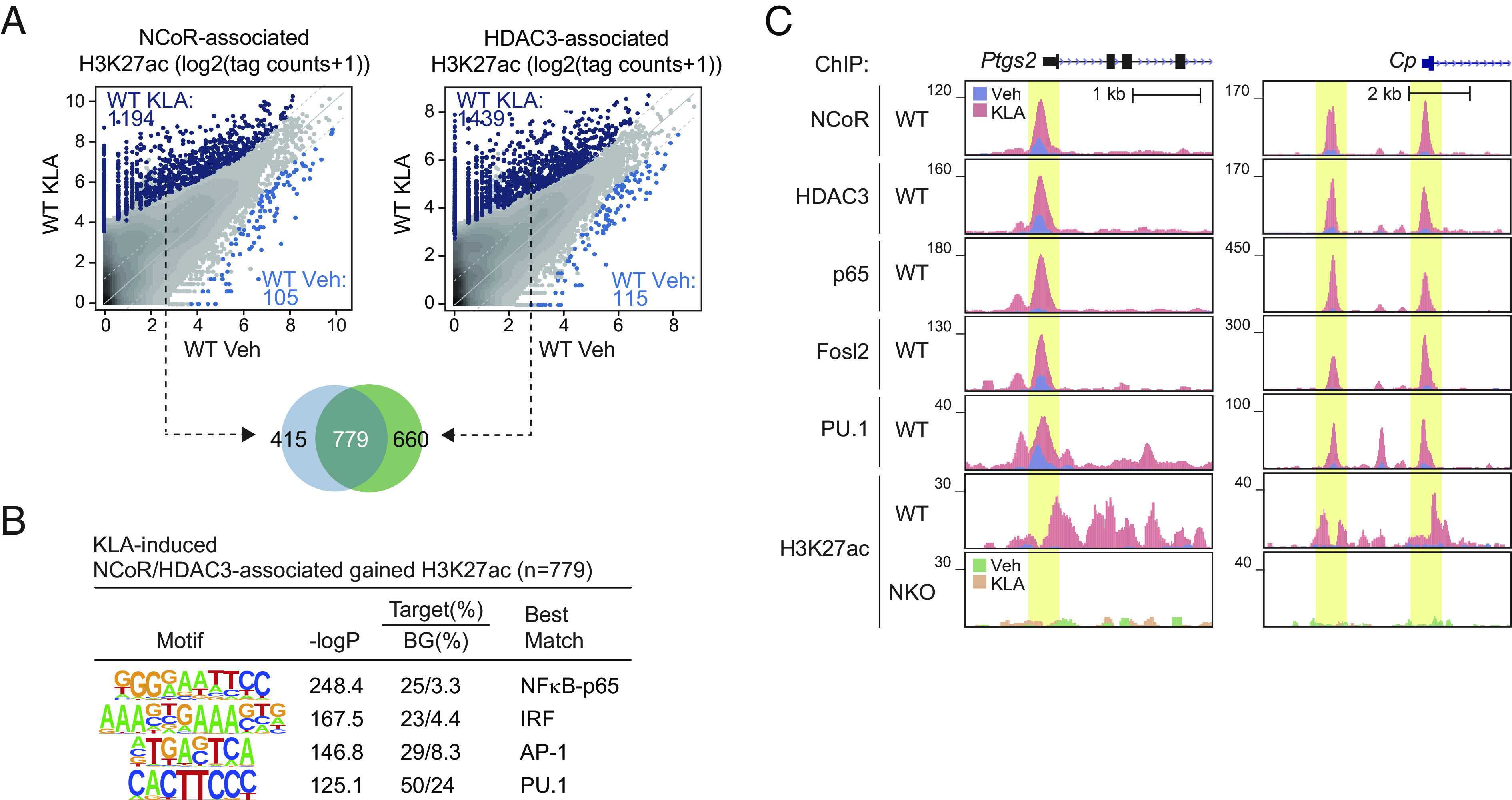
TLR4 signaling induces recruitment of NCoR/HDAC3 complex to enhancers and promoters. (*A*) Scatter plot of distal NCoR- (*Left*) or HDAC3- (*Right*) associated H3K27ac in WT at Veh vs. WT at KLA. KLA-induced NCoR- or HDAC3-associated H3K27ac peaks (FDR < 0.05, FC > 2) are color-coded (light blue dots: significantly NCoR- or HDAC3-associated lost H3K27ac in WT at KLA, dark blue dots: significantly NCoR- or HDAC3-associated gained H3K27ac in WT at KLA). The overlap between NCoR-associated gained H3K27ac (n = 1,194) and HDAC3-associated gained H3K27ac (n = 1,439) is shown by the Venn diagram. (*B*) De novo motif enrichment analysis of KLA-induced NCoR and HDAC3-associated gained H3K27ac peaks (n = 779 in Fig. 2*A*) using a GC-matched genomic background. (*C*) Genome browser tracks of NCoR, HDAC3, p65, Fosl2, PU.1, and H3K27ac ChIP-seq peaks in the vicinity of the *Ptgs2* and *Cp* loci at Veh and KLA. Yellow shading: KLA-induced peaks. Please see also *SI Appendix*, Fig. S2.

To investigate mechanisms underlying the requirement of NCoR/HDAC3 complex for TLR4-induced gene expression, we used chromatin immunoprecipitation sequencing (ChIP-seq) to define the genome-wide locations of NCoR and HDAC3 in WT cells under control conditions and in response to KLA. KLA resulted in increases in the number of confident genomic binding sites of NCoR (*SI Appendix*, Fig. S2*A*) and HDAC3 (*SI Appendix*, Fig. S2*B*). In parallel, we performed ChIP-seq for H3K27ac under control and KLA conditions as a surrogate for promoter and enhancer activity (27). KLA treatment resulted in significant increases in H3K27ac at 2,247 locations (*SI Appendix*, Fig. S2*C*). To define the relationship between NCoR/HDAC3 binding and gain or loss of promoter/enhancer activity, we annotated NCoR and HDAC3 peaks for local H3K27ac (±500 bp) under control and KLA treatment conditions and determined their overlaps. In the case of KLA treatment, >770 of the genomic locations exhibiting constitutive or gained presumptive NCoR/HDAC3 complexes localized to promoters and putative enhancers that exhibited a >2-fold increase in H3K27ac, whereas less than 100 of such regions were associated with the expected loss of H3K27ac (Fig. 2*A*). NCoR/HDAC3-associated peaks gaining H3K27ac were most highly enriched for NFκB-p65, AP-1, and IRF recognition motifs, along with the motif for the macrophage lineage determining transcription factor PU.1 (Fig. 2*B*). Signal-dependent recruitment of NCoR/HDAC3 to sites of gained H3K27ac is exemplified at the promoters or enhancers associated with *Ptgs2* and *Cp* (Fig. 2*C*). ChIP-sequencing experiments confirmed KLA-dependent increases in the binding of NFκB-p65 and PU.1 at sites of NCoR/HDAC3 binding (Fig. 2*C*). To trace AP-1 factors, we performed ChIP-sequencing for Fosl2 (Fig. 2*C*), which we previously found to be induced at RANKL-induced sites of NCoR/HDAC3/PGC1β interaction (19), and which exhibited the most highly correlated DNA binding profile with KLA-induced binding of NCoR in comparison to cJun, cFos, and ATF3, illustrated for Fosl2 and ATF3 in *SI Appendix*, Fig. S2*D*.

### HDAC3 Activity Leads to TLR4-Induced H3K27 Acetylation.

**Fig. 3.**
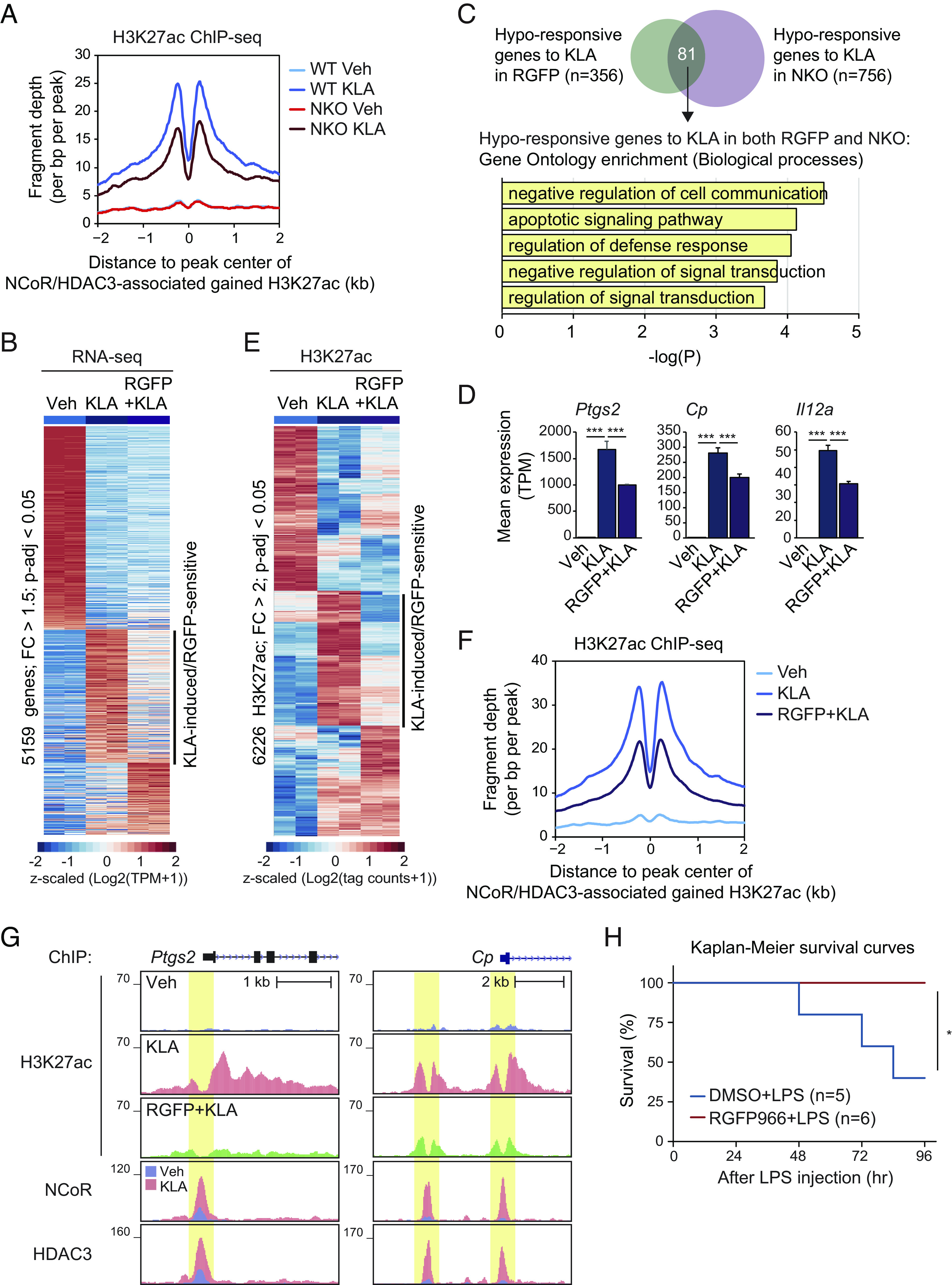
HDAC3 activity leads to TLR4-induced H3K27 acetylation. (*A*) Normalized distribution of H3K27ac tag density in WT and NKO at the vicinity of NCoR and HDAC3-associated gained H3K27ac peaks in WT at KLA (n = 779 in Fig. 2*A*). (*B*) Heatmap of differential gene expression (FC > 1.5, *P*-adj < 0.05) in WT cells treated with the combination of RGFP966 with KLA. (*C*) The overlap between hyporesponsive genes to KLA in RGFP966 treatment and in NKO (Fig. 1*B*) is shown by the Venn diagram. The significant gene ontology terms associated with the hyporesponsive genes (n = 81) are shown. (*D*) Bar plots for expression of *Ptgs2*, *Cp,* and *Il12a*. The significance symbols indicate statistical significance, ***P-adj < 0.001 reported by DESeq2 using the Benjamini–Hochberg method for the multiple-testing correction. (*E*) Heatmap of differential H3K27ac ChIP-seq IDR peaks associated with ATAC-seq IDR peaks at Veh in a 1,000-bp window (FC > 2, *P*-adj < 0.05). (*F*) Normalized distribution of H3K27ac tag density in Veh, KLA, and RGFP966 + KLA conditions at the vicinity of NCoR and HDAC3-associated gained H3K27ac peaks in WT at KLA (n = 779 in Fig. 2*A*). (*G*) Genome browser tracks of H3K27ac, NCoR, and HDAC3 ChIP-seq peaks in the vicinity of the *Ptgs2* and *Cp* loci in BMDMs at Veh, KLA, and the combination of RGFP966 with KLA. Yellow shading: KLA-induced/RGFP966-sensitive peaks. (*H*) Kaplan–Meier survival curves of wild-type mice subjected to 6 mg/kg LPS by intraperitoneal injection with pretreatment of either vehicle control (10% DMSO) or 10 mg/kg RGFP966. *P < 0.05 calculated using a Mantel–Cox test. Please see also *SI Appendix*, Fig. S3.

To investigate whether there was a causal relationship between NCoR/HDAC3 binding and increased H3K27ac, we performed ChIP-seq for H3K27ac in NCoR-deficient macrophages. This revealed marked reduction in H3K27ac at NCoR/HDAC3 binding sites exhibiting gain of H3K27ac in response to KLA (Figs. 2*C* and 3*A*). In parallel, we investigated the role of HDAC3 histone deacetylase activity by examining the consequences of the HDAC3-specific inhibitor RGFP966 (28) on KLA-dependent gene expression and histone acetylation. Treatment of BMDMs with the combination of KLA and RGFP966 resulted in an attenuated activation of a large fraction of genes (Fig. 3*B*), consistent with prior findings in RAW 264.7 macrophages (16). This set included 81 hyporesponsive genes in the context of the NCoR knockout (Fig. 3 *C* and *D*) which were significantly enriched for functional annotation related to apoptotic signaling pathway and regulation of defense response (Fig. 3*C*). Similarly, KLA-induced H3K27ac peaks were significantly reduced in the presence of RGFP966 (Fig. 3*E*) which associated with NCoR/HDAC3-bound active regions (Fig. 3 *F* and *G*). Further, analysis of prior RNA-seq data of LPS-treated macrophages from mice in which the deacetylase activating domain of NCoR/SMRT was mutated to preclude deacetylase activity of HDAC3 (NSDAD) (*SI Appendix*, Fig. S3*A*) or the deacetylase activity of HDAC3 was directly abolished by an inactivating mutation (F298Y) (*SI Appendix*, Fig. S3*B*) (14) revealed an overlapping profile of compromised TLR4-dependent gene expression in RGFP966-treated (Fig. 3*D*) or NCoR KO (Fig. 1*C*) macrophages. Intriguingly, RGFP966 treatment completely prevented mortality resulting from intraperitoneal injection of LPS (Fig. 3*H*) which is consistent with the previous report that RGFP966 suppressed LPS-induced NFκB-p65 transcriptional activity in macrophages (16). Collectively, these results provide evidence that the NCoR/HDAC3 complex is paradoxically required for histone acetylation and gene activation in response to TLR4 signaling.

### Signal-Specific Assembly and Function of NCoR/HDAC3/PGC1β Complexes.

**Fig. 4.**
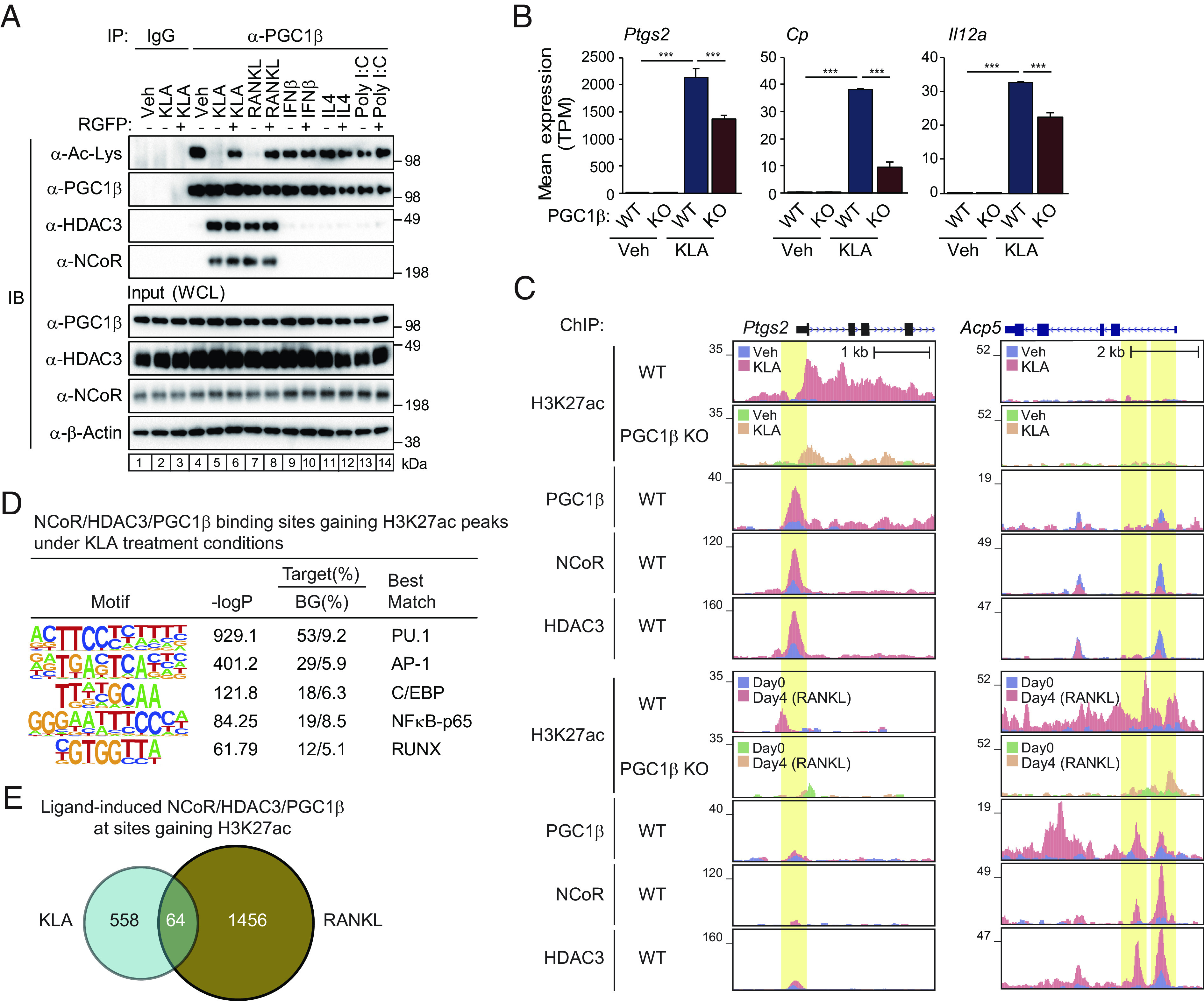
Signal-specific assembly and function of NCoR/HDAC3/PGC1β complexes. (*A*) Immunoblot (IB) analysis showing acetylated PGC1β and interaction of PGC1β with NCoR/HDAC3 complex. BMDMs were treated with or without each ligand in the presence or absence of RGFP966, and then, the whole-cell lysates were subjected to immunoprecipitation (IP) using anti-PGC1β antibody and IB analysis with antiacetylated lysine, PGC1β, HDAC3, or NCoR antibody. Uncropped images of the blots are shown in *SI Appendix*, Fig. S7. (*B*) Bar plots for expression of *Ptgs2*, *Cp,* and *Il12a* in *Pgc1b^f/f^* (WT) and *Pgc1b^f/f^ LysM-Cre* (KO) BMDMs treated with or without KLA. The significance symbols indicate statistical significance, ***P-adj < 0.001 reported by DESeq2 using the Benjamini–Hochberg method for the multiple-testing correction. (*C*) Genome browser tracks of H3K27ac, PGC1β, NCoR, and HDAC3 ChIP-seq peaks in the vicinity of the *Ptgs2* and *Acp5* loci. Yellow shading: lost H3K27ac by PGC1β KO at KLA- (*Top*) or RANKL- (*Bottom*) induced NCoR, HDAC3 and PGC1β binding regions. (*D*) De novo motif enrichment analysis of NCoR/HDAC3/PGC1β binding sites gaining H3K27ac peaks under KLA treatment conditions (n = 1,486 in *SI Appendix*, Fig. S4*D*) using a GC-matched genomic background. (*E*) The overlap between KLA- and RANKL-induced NCoR/HDAC3/PGC1β peaks at sites gaining H3K27ac peaks is shown by the Venn diagram. Please see also *SI Appendix*, Fig. S4.

In our prior studies, treatment of macrophages with RANKL promoted the interaction of NCoR/HDAC3 complexes with PGC1β, converting NCoR from a corepressor to a coactivator of gene transcription (19). The requirement of NCoR for full activation of gene expression by RANKL and KLA, but not for activation of gene expression by poly I:C, IFNβ and IL4 (Fig. 1*A*), suggested signal specificity in the mechanisms promoting NCoR/HDAC3/PGC1β interaction. To investigate this possibility, we treated BMDMs with vehicle or each of these ligands, immunoprecipitated PGC1β, and evaluated its state of acetylation and interaction with NCoR and HDAC3 by immunoblotting (Fig. 4*A*). Treatment of BMDMs with KLA or RANKL resulted in interaction of PGC1β with NCoR/HDAC3 and its deacetylation (Fig. 4*A*, compare lanes 4 and 5 or 7), in agreement with prior studies (19). The HDAC3-specific inhibitor RGFP966 prevented KLA- and RANKL-dependent deacetylation of PGC1β, but not its interaction with NCoR/HDAC3 (Fig. 4*A*). In contrast, stimulation of macrophages with IFNβ, IL4 or poly I:C did not promote interaction of PGC1β with NCoR or HDAC3 or its deacetylation (Fig. 4*A*, compare lanes 4 and 9, 11 or 13). These findings indicated that the assembly of NCoR/HDAC3/PGC1β complexes is signal-specific.

To establish the dependence of TLR4-induced gene expression on PGC1β, we crossed *LysM-Cre* mice to homozygous floxed PGC1β (*Pgc1b^f/f^* ) mice (29) (*SI Appendix*, Fig. S4*A*), and used BMDMs for RNA-seq and H3K27ac ChIP-seq studies. The expression of NCoR/HDAC3-dependent KLA-induced genes (e.g., *Ptgs2*, *Cp,* and *Il12a*) was reduced in BMDMs from *Pgc1b^f/f^ LysM-Cre* (PGC1β KO) mice (Fig. 4*B*). In accordance with these findings, the lack of PGC1β suppressed KLA-induced H3K27ac at NCoR/HDAC3/PGC1β-bound genomic regions, exemplified at the *Ptgs2* and *Cp* genes (Fig. 4*C* and *SI Appendix*, Fig. S4*B*). To investigate whether PGC1β colocalizes with NCoR and HDAC3 at KLA-activated enhancers and promoters, we evaluated its genome-wide locations under control and KLA treatment conditions. ChIP-sequencing experiments revealed ~33,000 high-confidence PGC1β binding sites in BMDMs under control conditions (*SI Appendix*, Fig. S4*C*). KLA treatment led to increased binding at >12,300 sites (*SI Appendix*, Fig. S4*C*). Intersection of PGC1β binding sites under KLA treatment conditions with the 2,247 regions of open chromatin exhibiting >twofold increases in H3K27ac indicated that 1,486 (66%) were also occupied by the combination of NCoR and HDAC3 (*SI Appendix*, Figs. S2*C* and S4*D*). These locations were enriched for PU.1, AP-1, CCAAT-enhancer-binding protein (C/EBP) and NFκB-p65 recognition motifs (Fig. 4*D*). In contrast, this combination was only observed at 648 (32%) of the 2,023 regions of open chromatin exhibiting >twofold decreases in H3K27ac (*SI Appendix*, Figs. S2*C* and S4*E*), which were enriched for binding sites for PU.1, AP-1 and C/EBP but not NFκB-p65 (*SI Appendix*, Fig. S4*F*). These findings are consistent with TLR4-dependent assembly of PGC1β/NCoR/HDAC3 complexes with coactivator function at specific genomic loci, analogous to those previously described in response to RANKL (19).

Although RANKL and KLA both promote the assembly of NCoR/HDAC3/PGC1β complexes that localize to sites of NFκB binding, the genes that are activated by these ligands are mostly different, promoting osteoclast development or innate immune responses, respectively. To investigate whether these complexes are directed to different genomic locations in a signal-specific manner, we compared their binding sites at locations gaining H3K27ac in response to RANKL or KLA. Interestingly, although the NCoR/HDAC3/PGC1β complexes under KLA or RANKL treatment conditions commonly existed on active gene elements enriched for AP-1 and NFκB-p65 binding motifs (Fig. 4*D*) (19), only ~10% of these regions were in common (Fig. 4*E*). Examples of RANKL-specific induction of NCoR/HDAC3/PGC1β complexes are provided by *Acp5* and *Ocstamp*, in contrast to the KLA-specific induction at *Ptgs2* and *Cp* (Fig. 4*C* and *SI Appendix*, Fig. S4*B*). De novo motif enrichment analysis of KLA-specific locations in comparison to RANKL-specific locations revealed strong enrichment of IRF1 motifs in the KLA-specific locations (*Top*, *SI Appendix*, Fig. S4 *G*), consistent with activation of IRF transcription factors in response to TLR4 signaling but not RANK signaling. Conversely, BATF/AP-1 motif was strongly enriched in RANKL-specific locations, suggesting critical roles of ATF/AP-1 factors in modulating NCoR/HDAC3/PGC1β complexes downstream of RANK (*Bottom*, *SI Appendix*, Fig. S4 *G*).

### NCoR/HDAC3/PGC1β Complexes Contain the Histone Acetyltransferase p300.

**Fig. 5.**
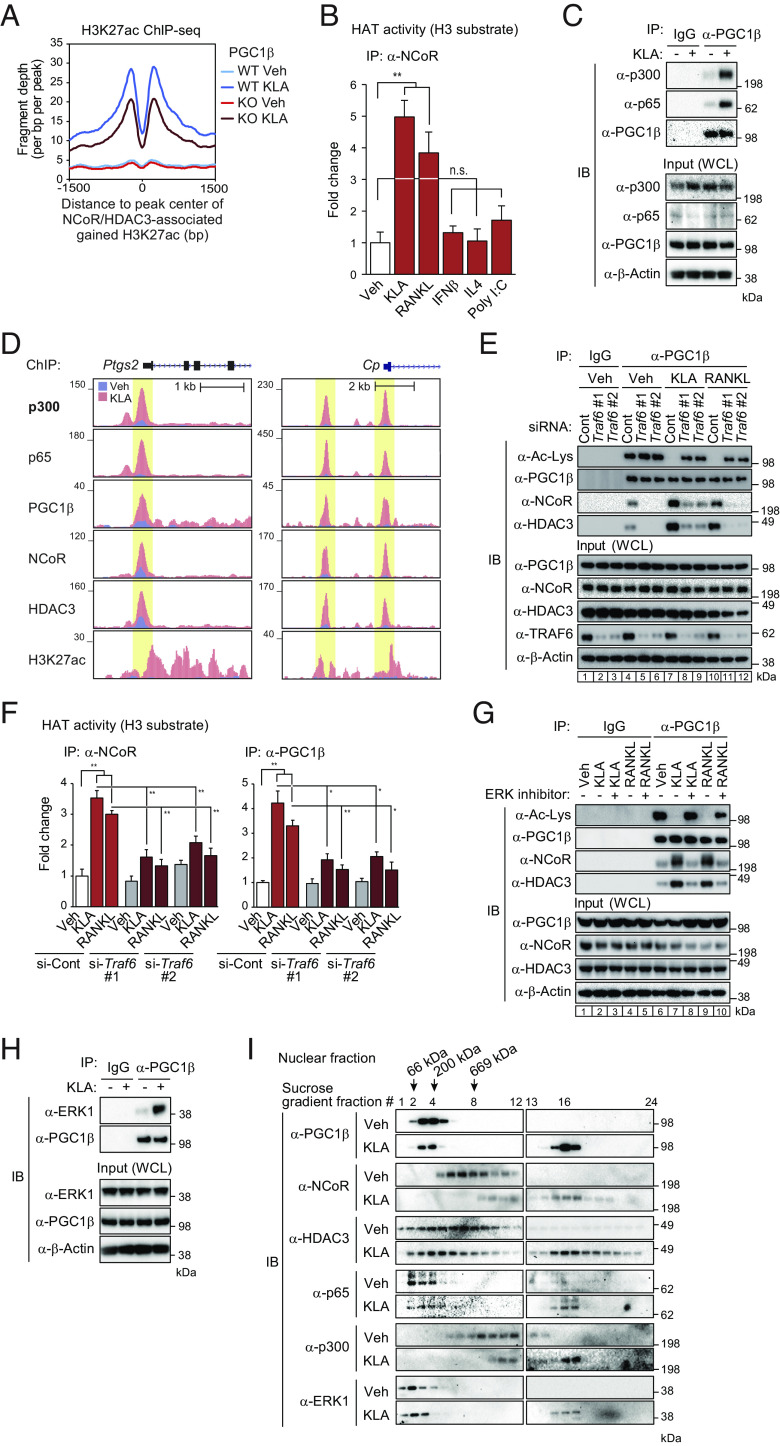
TRAF6-ERK1 signaling is required for PGC1β interaction with NCoR/HDAC3 complex. (*A*) Normalized distribution of H3K27ac ChIP-seq tag density in *Pgc1b^f/f^* (WT) and *Pgc1b^f/f^ LysM-Cre* (KO) BMDMs at the vicinity of NCoR and HDAC3-associated gained H3K27ac peaks in WT under KLA conditions (n = 779 in Fig. 2*A*). (*B*) Histone acetyltransferase (HAT) activity of immunoprecipitated NCoR protein in whole-cell lysates from BMDMs treated with or without each ligand was measured in the presence of acetyl-CoA and histone H3 substrate. Data are mean ± SD (n = 3 biological replicates). Student’s *t* test was performed for comparisons. **P < 0.01 was considered statistically significant. (*C*) Immunoblot (IB) analysis showing the interaction of PGC1β with p300 or NFκB-p65 in BMDMs treated with or without KLA. The whole-cell lysates were subjected to immunoprecipitation (IP) using anti-PGC1β antibody and IB analysis with anti-p300, p65, or PGC1β antibody. (*D*) Genome browser tracks of p300, p65, PGC1β, NCoR, HDAC3, and H3K27ac ChIP-seq peaks in the vicinity of the *Ptgs2* and *Cp* loci in BMDMs at Veh and KLA. Yellow shading: KLA-induced peaks. (*E*) IB analysis showing acetylated PGC1β and interaction of PGC1β with NCoR/HDAC3. Control or *Traf6* siRNA-transduced BMDMs were treated with or without KLA or RANKL, and then, the whole-cell lysates were subjected to IP using anti-PGC1β antibody and IB analysis with antiacetylated lysine, PGC1β, NCoR, or HDAC3 antibody. (*F*) HAT activity of immunoprecipitated NCoR (*Left*) or PGC1β (*Right*) protein in whole-cell lysate from control or *Traf6* siRNA-transduced BMDMs treated with or without KLA or RANKL was measured in the presence of acetyl-CoA and histone H3 substrate. Data are mean ± SD (n = 3 biological replicates). ANOVA was performed followed by Tukey’s post hoc comparison. *P < 0.05 and **P < 0.01 were considered statistically significant. (*G*) IB analysis showing acetylated PGC1β and interaction of PGC1β with NCoR/HDAC3. BMDMs were treated with or without KLA or RANKL in the presence or absence of ERK inhibitor (LY3214996), and then, the whole-cell lysates were subjected to IP using anti-PGC1β antibody and IB analysis with antiacetylated lysine, PGC1β, NCoR, or HDAC3 antibody. (*H*) IB analysis showing the interaction of PGC1β with ERK1 in BMDMs treated with or without KLA. The whole-cell lysates were subjected to IP using anti-PGC1β antibody and IB analysis with anti-ERK1 or PGC1β antibody. (*I*) 10 to 30% sucrose density gradient centrifugation was performed on nuclear fractions from BMDMs treated with or without KLA. All fractions (1 to 24, top to bottom) were subjected to IB analysis with anti-PGC1β, NCoR, HDAC3, p65, p300, or ERK1 antibody. The molecular weight standards are indicated at the top of the panel; 66 kDa, bovine serum albumin; 200 kDa, β-amylase; 669 kDa, thyroglobulin. Uncropped images of the blots (*C*, *E*, *G*, *H*, and *I*) are shown in *SI Appendix*, Figs. S7 and S8. Please see also *SI Appendix*, Fig. S5.

The requirement of PGC1β for KLA-induced histone acetylation exemplified at the *Ptgs2* and *Cp* loci (Fig. 4*C* and *SI Appendix*, Fig. S4*B*) and at a genome-wide level (Fig. 5*A*) suggested that NCoR/HDAC3/PGC1β complexes acquire a histone acetyltransferase (HAT) activity in a signal-dependent manner. Consistent with this, PGC1β complexes acquired HAT activity in response to KLA and RANKL, but not IFNβ, IL4 or poly I:C (Fig. 5*B*). In agreement with these findings, KLA treatment markedly increased the interaction of PGC1β with the histone acetyltransferase p300 in conjunction with the p65 component of NFκB (Fig. 5*C*). The finding of HAT activity and p300 in NCoR complexes raised the question as to whether p300 is colocalized with these complexes on DNA. Genome-wide location analyses of p300 in BMDMs indicated KLA treatment led to increased binding at >29,000 sites (*SI Appendix*, Fig. S5*A*), whereas RANKL treatment led to increased binding at >17,000 sites (*SI Appendix*, Fig. S5*B*), which were significantly enriched for binding motifs for AP-1 and NFκ-p65 (*SI Appendix*, Fig. S5 *C* and *D*). Of the genomic regions at which KLA or RANKL treatment induced NCoR/HDAC3/PGC1β and H3K27ac, more than 85% were also bound by p300 (*SI Appendix*, Fig. S5 *E* and *F*), exemplified by *Ptgs2* and *Cp* (Fig. 5*D*). Collectively, these findings indicate that among the ligands tested, KLA and RANKL selectively promote the conversion of NCoR/HDAC3 corepressor complexes to NCoR/HDAC3/PGC1β/p300 coactivator complexes that are required for acetylation of H3K27.

### TRAF6-ERK1 Signaling Is Required for PGC1β Interaction with NCoR/HDAC3 Complex.

A distinguishing feature of signaling downstream of TLR4 and RANK in comparison to TLR3, IL4, and IFNβ in myeloid cells is the utilization of TRAF6 (30, 31). To investigate a potential role of TRAF6 in regulating the interaction of PGC1β with the NCoR/HDAC3 complex, independent short interfering RNAs (siRNAs) specifically targeting *Traf6* were transduced into BMDMs to knockdown TRAF6 expression. Notably, the ligand-induced complex formation of PGC1β with NCoR/HDAC3 was suppressed by TRAF6 knockdown in concert with the reduction of PGC1β deacetylation (Fig. 5*E*, compare lanes 7 and 8/9, or 10 and 11/12). Consistent with this, TRAF6 knockdown also suppressed the ligand-induced HAT activities associated with NCoR/HDAC3/PGC1β complexes (Fig. 5*F*). TLR4 agonists induce the ubiquitination of TRAF6, which has been reported to be essential for MAPK/ERK activation (32) and RANKL-induced osteoclast differentiation via TRAF6 has been shown to be dependent on ERK1/2 and NFκB activation (33). To investigate a potential role of ERK activation in NCoR/HDAC3/PGC1β complex formation, PGC1β was immunoprecipitated in BMDMs treated with or without ERK inhibitor and subject to immunoblotting. ERK inhibitor suppressed the interaction between PGC1β and NCoR/HDAC3 accompanied by reduced deacetylation in PGC1β (Fig. 5*G*, compare lanes 7 and 8, or 9 and 10), suggesting TRAF6-ERK signaling is necessary to establish NCoR/HDAC3/PGC1β complex for transcriptional activation. Interestingly, PGC1β interacted with ERK1 in response to KLA treatment (Fig. 5*H*).

To examine the properties of NCoR, HDAC3, and PGC1β protein complexes in nuclear fractions of macrophages treated with or without KLA, we performed sucrose density gradient centrifugation and analyzed gradient fractions by immunoblotting. In vehicle conditions, PGC1β was primarily found in low molecular weight fractions 2 to 5. In contrast, both NCoR and HDAC3 were observed in fractions 5 to 12, consistent with previously described NCoR/HDAC3 corepressor complexes (2–4). Following treatment with KLA for 6 h, substantial portions of NCoR, HDAC3, and PGC1β shifted to higher-molecular-weight fractions, with cosedimentation of all three proteins in fractions 15 to 17 (Fig. 5*I*), consistent with the signal-dependent induction of a distinct stable NCoR/HDAC3/PGC1β complex (Fig. 4*A*). The protein size of the NCoR/HDAC3/PGC1β complex established by TLR4 signaling is estimated to be ~2 MDa, consistent with that observed in response to RANK signaling (19). In addition, significant portions of NFκB-p65, p300, and ERK1 cosedimented with NCoR/HDAC3/PGC1β complexes in fraction 15-17 in response to KLA treatment (Fig. 5*I*), consistent with KLA-dependent interaction of PGC1β with p65, p300 and ERK1 (Fig. 5 *C* and *H*).

### ERK Activity Is Required for KLA-Induced NCoR Recruitment to Active Genes.

**Fig. 6.**
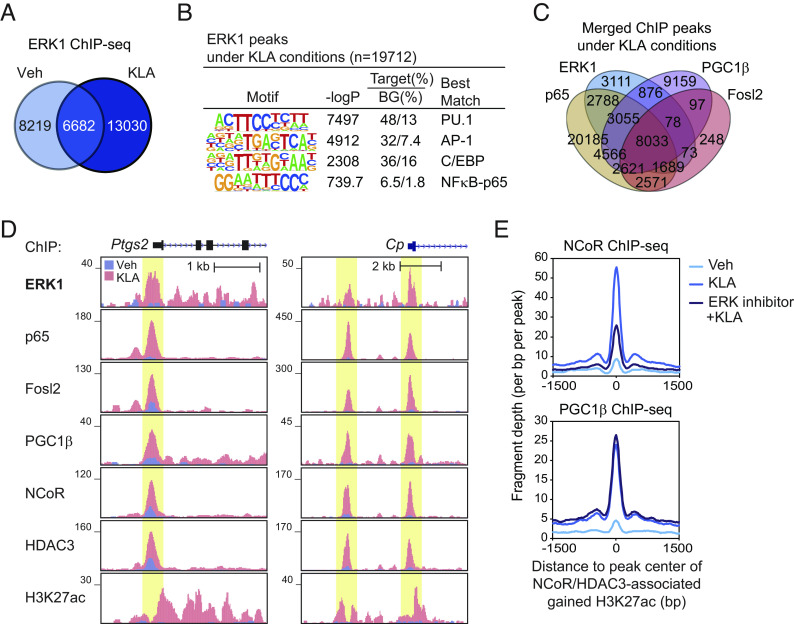
ERK activity is required for KLA-induced NCoR recruitment to active genes. (*A*) The overlap between IDR-defined ERK1 ChIP-seq peaks at Veh and KLA is shown by the Venn diagram. (*B*) De novo motif enrichment analysis of ERK1 peaks under KLA conditions (n = 19,712 in Fig. 6*A*) using a GC-matched genomic background. (*C*) The overlap between IDR-defined ERK1, PGC1β, p65, and Fosl2 ChIP-seq peaks under KLA conditions is shown by the Venn diagram. (*D*) Genome browser tracks of ERK1, p65, Fosl2, PGC1β, NCoR, HDAC3, and H3K27ac ChIP-seq peaks in the vicinity of the *Ptgs2* and *Cp* loci in WT at Veh and KLA. Yellow shading: KLA-induced peaks. (*E*) Normalized distribution of NCoR (*Top*) or PGC1β (*Bottom*) ChIP-seq tag density in BMDMs treated with or without KLA in the presence or absence of ERK inhibitor (LY3214996) at the vicinity of NCoR and HDAC3-associated gained H3K27ac peaks in WT under KLA conditions (n = 779 in Fig. 2*A*). Please see also *SI Appendix*, Fig. S6.

The finding of ERK1 in stable NCoR/HDAC3/PGC1β complexes (Fig. 5 *H* and *I*) raised the question as to whether it is retained in these complexes on DNA. Genome-wide location analyses of ERK1 in BMDMs indicated KLA treatment led to increased binding at >13,000 sites (Fig. 6*A*), whereas RANKL treatment led to increased binding at >9,600 sites (*SI Appendix*, Fig. S6*A*), which were significantly enriched for binding motifs for AP-1 and NFκB-p65 (Fig. 6*B* and *SI Appendix*, Fig. S6*B*). ERK1, PGC1β, NFκB-p65, and the AP-1 factor Fosl2 were colocalized at more than 8,000 (Fig. 6*C*) and 2,700 (*SI Appendix*, Fig. S6*C*) genomic regions under KLA and RANKL conditions, respectively. 52% of the 2247 regions (*SI Appendix*, Figs. S2*C* and S6*D*) and 19% of the 1,525 regions (*SI Appendix*, Fig. S6*E*) (19) of open chromatin exhibiting >twofold increases in H3K27ac under KLA and RANKL treatments, respectively showed the combination of ERK1 with NCoR/HDAC3/PGC1β, consistent with cosedimentation of all four proteins in high molecular weight fractions in a signal-dependent manner (Fig. 5*I*). Examples of these patterns are shown for *Ptgs2* and *Cp* under vehicle and KLA conditions (Fig. 6*D*). To investigate whether ERK activity would affect DNA binding of NCoR or PGC1β, we performed ChIP-seq for NCoR or PGC1β in BMDMs treated with KLA in the presence of ERK inhibitor. This revealed marked reduction in NCoR (*Top*, Fig. 6 *E*) depending on the treatment of ERK inhibitor but not PGC1β (*Bottom*, Fig. 6 *E*) at NCoR/HDAC3 binding sites exhibiting gain of H3K27ac in response to KLA, suggesting that KLA-induced recruitment of NCoR to active genes requires ERK activity.

### TRAF6-TBK1 Cascade Accelerates HDAC3-Dependent PGC1β Deacetylation.

**Fig. 7.**
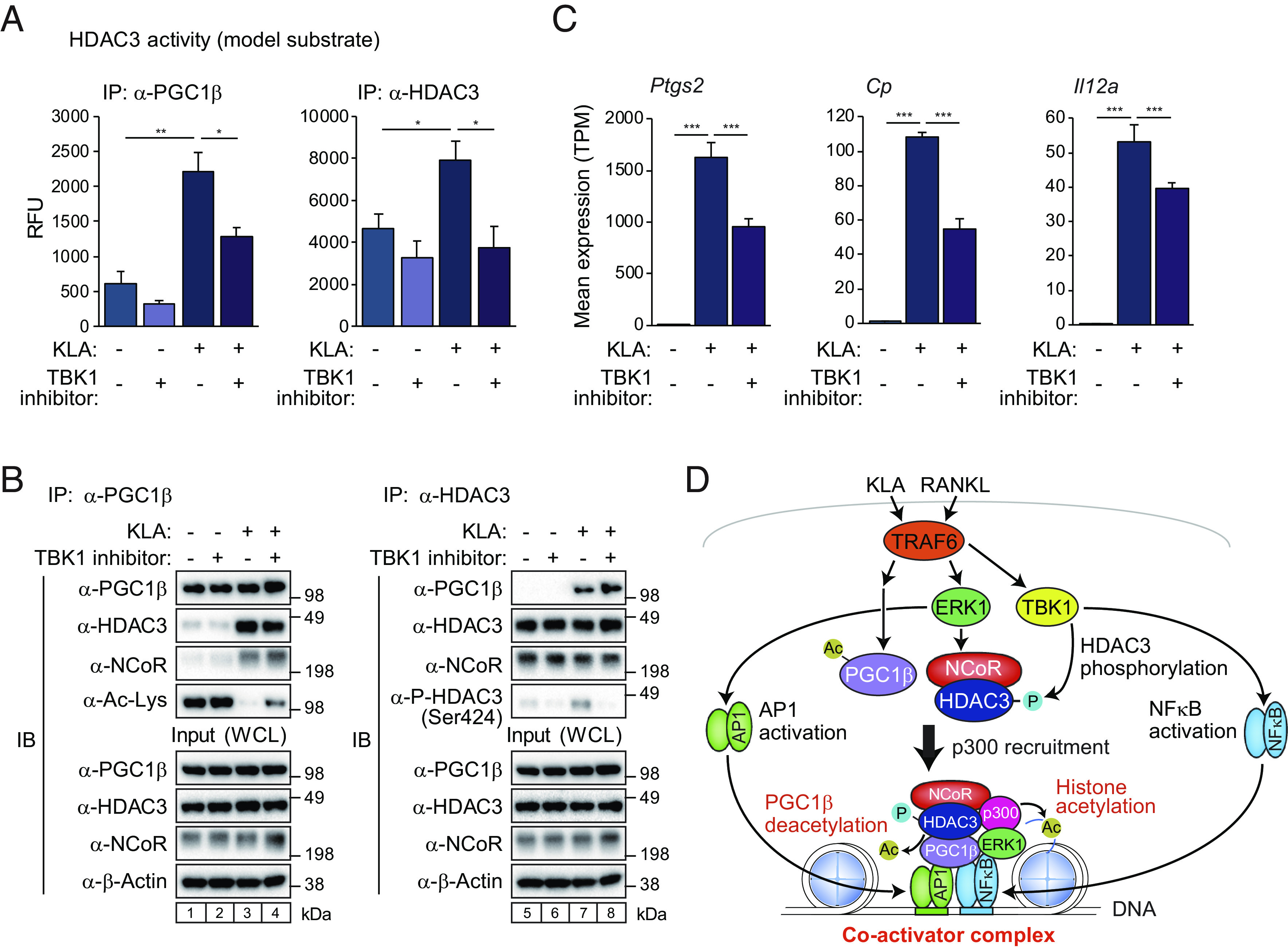
TRAF6-TBK1 cascade accelerates HDAC3-dependent PGC1β deacetylation. (*A*) HDAC3 deacetylase activity of immunoprecipitated PGC1β (*Left*) or HDAC3 (*Right*) protein in whole-cell lysate from BMDMs treated with or without KLA in the presence or absence of TBK1 inhibitor (GSK8612) was measured by the fluorescent signal of the enzymatically cleaved model substrates. Data are mean ± SD (n = 3 biological replicates). ANOVA was performed followed by Tukey’s post hoc comparison. *P < 0.05 and **P < 0.01 were considered statistically significant. (*B*) Immunoblot (IB) analysis showing acetylated PGC1β, phosphorylated HDAC3, and interaction of PGC1β with NCoR/HDAC3. BMDMs were treated with or without KLA, and then, the whole-cell lysates were subjected to immunoprecipitation (IP) using anti-PGC1β (*Left*) or HDAC3 (*Right*) antibody and IB analysis with antiacetylated lysine, phosphorylated HDAC3 (Ser424), PGC1β, NCoR, or HDAC3 antibody. Uncropped images of the blots are shown in *SI Appendix*, Fig. S9. (*C*) Bar plots for expression of *Ptgs2*, *Cp,* and *Il12a* in BMDMs treated with or without KLA in the presence or absence of TBK1 inhibitor (GSK8612). The significance symbols indicate statistical significance, ***P-adj < 0.001 reported by DESeq2 using the Benjamini–Hochberg method for the multiple-testing correction. (*D*) A schematic model. See the *Discussion* for details.

TBK1 is an important signaling hub downstream of TRAF6, which plays a pivotal role in innate immune response and inflammation through the NFκB signaling pathway (34, 35). It has been reported that TBK1 directly phosphorylates HDAC3 at Ser424 and promotes the deacetylase activity (36). To understand the relationship of TBK1 to HDAC3 activity in the presence or absence of TLR4 stimulation, BMDMs were treated with TBK1 inhibitor for 12 h before KLA stimulation, and then immunoprecipitated PGC1β or HDAC3 protein was subject to HDAC3 activity assays using a low-molecular-weight nonhistone substrate. KLA-induced HDAC3 activity in immunoprecipitated PGC1β protein was partially suppressed by TBK1 inhibitor (*Left*, Fig. 7 *A*). On the other hand, the activity of immunoprecipitated HDAC3 protein increased in the presence of KLA and TBK1 inhibitor completely reduced HDAC3 activity up to basal condition (*Right*, Fig. 7 *A*). Interestingly, although TBK1 inhibitor did not affect KLA-induced interaction of PGC1β with NCoR/HDAC3 complex, KLA-induced PGC1β deacetylation was partially diminished by TBK1 inhibitor (*Left*, Fig. 7 *B*, compare lanes 3 and 4). TBK1 inhibitor abolished KLA-induced HDAC3 phosphorylation (*Right*, Fig. 7 *B*, compare lanes 7 and 8), in agreement with the observation that TBK1 inhibitor suppressed the activity of immunoprecipitated HDAC3 protein (*Right*, Fig. 7 *A*). Furthermore, treatment of BMDMs with the combination of KLA and TBK1 inhibitor resulted in an attenuated activation of NCoR/HDAC3/PGC1β complex target genes such as *Ptgs2*, *Cp,* and *Il12a* (Fig. 7*C*), consistent with reduced deacetylation and activation of PGC1β (*Left*, Fig. 7 *B*).

## Discussion

In concert, these studies identify a TLR4/TRAF6-dependent signaling pathway that converts NCoR from a corepressor of nuclear receptors to a coactivator of NFκB target genes that mediate inflammatory responses. Physiologic significance of this pathway is supported by the observation that myeloid-specific NCoR KO mice and mice treated with the HDAC3-specific inhibitor RGFP966 exhibit reduced mortality in response to LPS-induced septic shock in vivo. On the other hand, a pan-HDAC inhibitor (SAHA) enhanced toxicity in LPS-injected mice (14), which may reflect the fact that SAHA is more effective at blocking other class I HDACs (HDAC1, 2, and/or 8) rather than HDAC3 (37). The present findings also provide an additional mechanism to explain the insulin-sensitive phenotype of myeloid-specific NCoR KO mice beyond derepression of LXR target genes that promote the synthesis of anti-inflammatory fatty acids (12).

Analysis of the responses of wild type and NCoR KO macrophage to KLA, poly I:C, IL4, IFNβ and RANKL, revealed that NCoR was selectively required for normal TLR4- and RANK-induced gene expression. This pattern of ligand dependency was traced to the common utilization of TRAF6 by TLR4 and RANK. We propose a model in which TLR4 or RANK activation of TRAF6 initiates a bifurcated kinase cascade that coordinately regulates distinct steps mediating corepressor to coactivator conversion. The TRAF6-ERK signal establishes NCoR/HDAC3/PGC1β complex formation and recruitment of the p300 histone acetyltransferase. In parallel, the TRAF6-TBK1 signal enhances HDAC3 activity required for PGC1β deacetylation (Fig. 7*D*). Interaction of PGC1β with the NCoR/HDAC3 complex prevents HDAC3 from deacetylating H3K27ac as we reported before (19). This combination of events results in local histone acetylation and gene activation, rather than histone deacetylation and gene repression.

Although TLR4 and RANK both couple to TRAF6 to drive NCoR/HDAC3/PGC1β complex formation, the genomic destinations of these factors associated with active enhancers and promoters are almost completely unique, consistent with the divergent programs of gene transcription driven by each receptor. De novo motif enrichment analyses of these signal-specific binding sites suggest that the induction of alternative collaborative transcription factors, including IRFs in the case of TLR4 and BATF factors downstream of RANK, are drivers of this specificity (*SI Appendix*, Fig. S4*G*). In addition, previous studies of RANK-induced complex formation identified essential roles of noncoding RNAs including *Dancr* and *Rnu12* (19). It will be of interest to determine whether distinct noncoding RNAs mediate NCoR/HDAC3/PGC1β complex formation in response to TLR4 signaling or influence the genomic binding sites of these complexes.

We also note that the present findings are consistent with the previously reported NCoR-independent role of HDAC3 as a coactivator of TLR4-induced genes independent of its catalytic activity based on recruitment by ATF2 (14) and the presence of a fraction of the nuclear pool of HDAC3 that is not associated with NCoR in complexes defined by density gradient centrifugation (Fig. 5*I*). Although the focus of the present studies was on the role of NCoR as a coactivator of TLR4 responsive genes, ~1,300 mRNAs exhibit increased expression in the context of KLA treatment of NCoR KO macrophages (Fig. 1*A*), consistent with a parallel role of TLR4 signaling in amplifying the corepressor activity of NCoR. Studies of the deacetylase-dependent roles of HDAC3 in repression of gene expression downstream of TLR4 implicated ATF3 as a transcription factor important for recruitment of NCoR/HDAC3 complexes mediating histone deacetylation (14). Based on the previously reported genome-wide localization of ATF3 in LPS-treated macrophages, we find that while there is some overlap with Fosl2 at sites of TLR4-induced NCoR binding, most of the ATF3 and Fosl2 binding sites are distinct (*SI Appendix*, Fig. S2*D*), consistent with divergent roles in mediating NCoR corepression and coactivation, respectively.

The present findings raise the possibility that NCoR/HDAC3/PGC1β complexes function as coactivators downstream of other pathways that utilize TRAF6 and/or ERK and have implications for the therapeutic applications of recently developed HDAC3-specific inhibitors in inflammatory and metabolic diseases. BRAF (also referred to as proto-oncogene B-Raf) encoding a serine/threonine kinase regulates the ERK signal transduction pathway. The V600E mutation constitutively activates ERK signaling, which has been identified in melanoma (38, 39), Langerhans cell histiocytosis (LCH) (40, 41), and hairy cell leukemia (42, 43). A recent study reported that the combination of BRAF inhibitors with a HDAC3 inhibitor is dramatically efficacious against various subtypes of melanoma with BRAF mutation (44), suggesting that NCoR/HDAC3 recruitment may contribute to oncogene activation under massive ERK activation in V600E-mutated cells. In LCH patients, giant osteoclast-like multinucleated cells have been observed in osseous and nonosseous lesions (45) and often lead to low bone density (46). Since NCoR/HDAC3/PGC1β complexes activate osteoclast-related genes (e.g., *Acp5* and *Ocstamp*) downstream of the RANK-TRAF6-ERK signal pathway, the constitutive activation of ERK signal in V600E-mutated cells may be associated with NCoR/HDAC3/PGC1β-induced osteoclastogenesis.

Based on the absence of PGC1β from a subset of NCoR and HDAC3 binding sites that exhibit gained H3K27ac in response to KLA or RANKL, this mechanism may not fully account for TRAF6-dependent coactivator activity of NCoR/HDAC3 complexes at all genomic locations. Additional nonhistone substrates of HDAC3 that promote gene activation could explain this discrepancy. However, functional coupling between regulatory elements can also result in coordinated changes in histone acetylation in the absence of local activities of specific transcription factors and coactivators. Thus, actions of NCoR/HDAC3/PGC1β coactivators at specific locations may exert dominant effects on patterns of histone acetylation across a cis-regulatory domain.

The mechanisms underlying the assembly and activation of NCoR/HDAC3/PGC1β coactivator complexes at specific enhancers and promoters remain to be established, but the presence of p65 in the induced complex strongly implicates this interaction in specifying the localization of the complex to sites of NFκB binding. Since NCoR/HDAC3 complexes function as conventional corepressors at LXR binding sites in the same cells, their functional conversion to coactivators at NFκB/AP-1 target genes may be established by local actions of ERK/TBK1 kinases within the nucleus. Consistent with this, the TLR4- or RANK-induced genomic binding pattern of ERK1 extensively colocalizes with NCoR/HDAC3/PGC1β complexes at sites exhibiting cobinding of NFκB and AP-1. It is thus striking that the bifurcated kinase pathway downstream of TRAF6 that regulates the assembly and function of NCoR/HDAC3/PGC1β pathway also regulates the parallel but convergent activities of NFκB (via TBK1) (47) and AP-1 (via ERK1) (48) (Fig. 7*D*). This mechanism thereby enables a highly integrated, signal-specific transcriptional response that may be prototypic for other signaling pathways.

## Materials and Methods

### BMDM Culture.

Bone marrow cells were obtained by flashing the tibia and femur from 8- to 12-wk-old C57BL/6 mice with RPMI 1640 (Corning) containing 10% FBS (Omega Biosciences), 1% penicillin/streptomycin+L-glutamine (Thermo Fisher Scientific) and lysed using red blood cell lysis buffer (Invitrogen). After counting, 20 million cells were seeded per 15 cm non-tissue culture plates in RPMI 1640 containing 10% FBS, 30% L929 cell conditioned laboratory-made media (as source of M-CSF), and 1% penicillin/streptomycin+L-glutamine. After 3 d, 16.7 ng/mL M-CSF (Shenandoah Biotechnology) was added into the media. After an additional 4 d, nonadherent cells were washed off, and adherent cells were harvested. 0.5 million cells per wells of 6-well plates (Sigma-Aldrich) for ATAC-seq and RNA-seq or 10 million cells per 15-cm tissue culture plates (Thermo Fisher Scientific) for ChIP-seq, immunoblotting, and immunoprecipitation were cultured in RPMI 1640 containing 10% FBS, 1% penicillin/streptomycin+L-glutamine, and 16.7 ng/mL M-CSF overnight. After the serum starvation in RPMI 1640 containing 1% FBS and 1% penicillin/streptomycin+L-glutamine for 12 h, cells were treated with 100 ng/mL KLA (Avanti Polar Lipids), 20 ng/mL IFNβ (PeproTech), 20 ng/mL IL4 (PeproTech), or 50 ng/mL Poly I:C (GE Healthcare Bioscience) for 6 h or 50 ng/mL RANKL (PeproTech) for 12 h. The treatment of 5 mM RGFP966, 100 nM LY3214996 (Selleckchem), or 10 mM GSK8612 (Selleckchem) was performed at the same time as the serum starvation.

### RNA Interference.

TRAF6 protein was depleted from BMDMs with synthetic small interference RNAs (siRNAs) targeting mouse *Traf6* (Dharmacon, Individual #1 J-042735-09, Individual #2 J-042735-10). siGENOME Non-Targeting siRNA #2 (Dharmacon, D-001210-02) was used for a negative control. Five million cells were transfected with each 30 nM siRNA using Lipofectamine™ RNAiMAX Transfection Reagent (Invitrogen) according to the manufacturer’s instructions with modifications as follows. Lipofectamine/siRNA complexes (240 pmol of siRNA plus 24 µL of Lipofectamine reagent) were formed in Opti-MEM (Thermo Fisher Scientific) for 15 min at room temperature and then added into cells and incubated for 5 min at room temperature. The cell mixtures were seeded in 10-cm tissue culture plates and cultured in RPMI 1640 containing 10% FBS, 1% penicillin/streptomycin+L-glutamine, and 16.7 ng/mL M-CSF. At 2 d after the siRNA transfection, serum starvation was performed in RPMI 1640 containing 1% FBS and 1% penicillin/streptomycin+L-glutamine for 12 h followed by stimulation of 100 ng/mL KLA or 50 ng/mL RANKL.

### Sucrose Density Gradient Centrifugation.

Twenty million BMDMs were homogenized in STM buffer (250 mM sucrose, 50 mM Tris-HCl (pH 7.4), 5 mM MgCl_2_, 1 mM PMSF, and 1× protease inhibitor cocktail) and incubated on ice for 30 min. After centrifugation at 800 g for 15 min at 4 °C, the pellet was washed with STM buffer twice and resuspended in 150 µL of lysis buffer [50 mM HEPES-KOH (pH 7.9), 150 mM NaCl, 1.5 mM MgCl_2_, 1% NP-40, 1 mM PMSF, and 1× protease inhibitor cocktail] by an ultrasound homogenizer (Bioruptor, Diagenode) for 10 min at 4 °C. The suspension was passed through an 18-gauge needle 15 times and sonicated by ultrasound homogenizer for 10 min at 4 °C. After centrifugation at 9,000 g for 30 min at 4 °C, the supernatant was used as nuclear fraction. Then, 150 µL of nuclear fraction at a concentration of 5.1 µg/µL was layered on top of a 12-mL 10 to 30% (w/v) continuous sucrose gradient [in buffer containing 50 mM HEPES-KOH (pH 7.9), 150 mM NaCl, 1.5 mM MgCl_2_, 1 mM PMSF, and 1× protease inhibitor cocktail]. Gradients were centrifuged at 32,000 rpm for 12 h at 4 °C in an SW41 Ti rotor (Beckman). Molecular weight standards (Sigma-Aldrich) in lysis buffer were run in parallel. Twenty-four fractions (500 µL each) were collected from each gradient, and 125 µL of 100% (w/v) trichloroacetic acid (Sigma-Aldrich) containing 4 mg/mL sodium deoxycholate was added to each fraction. After incubation on ice for 15 min, the samples were spun for 15 min, and the supernatant was removed. One mL of acetone (Sigma-Aldrich) was added to each pellet. Each sample was incubated for 10 min at room temperature and spun for 15 min. The supernatant was discarded, and the pellet was dissolved in NuPAGE™ LDS Sample Buffer (Thermo Fisher Scientific) with NuPAGE™ Sample Reducing Agent (Thermo Fisher Scientific). After heating at 95 °C for 5 min, the samples were subjected to SDS-PAGE.

## Supplementary Material

Appendix 01 (PDF)

## Data Availability

All sequencing data including RNA-seq, ChIP-seq, and ATAC-seq were deposited in the Gene Expression Omnibus (GEO) under accession number GSE247945 (49). All study data are included in the article and *SI Appendix*.
