# Supplementary material for: A TLR4/TRAF6-dependent signaling pathway mediates NCoR coactivator complex formation for inflammatory gene activation
Source: Proc Natl Acad Sci U S A. 2024 Jan 2;121(2):e2316104121. doi: 10.1073/pnas.2316104121 (PMC10786282; doi:10.1073/pnas.2316104121)
Supplement: Supplementary file 1 — Appendix 01 (PDF) [file pnas.2316104121.sapp.pdf]

PNAS

Supporting Information for

A TLR4/TRAF6-dependent signaling pathway mediates NCoR co-activator complex formation for inflammatory gene activation

Yohei Abe[a], Eric R. Kofman[a,b,c], Zhengyu Ouyang[a], Grisel Cruz-Becerra[d], Nathanael J. Spann[a], Jason S. Seidman[a], Ty D. Troutman[e,f], Joshua D. Stender[a], Havilah Taylor[g], Weiwei Fan[h], Verena M. Link[a,i], Zeyang Shen[a,j], Juro Sakai[k,l], Michael Downes[h], Ronald M. Evans[h], James T. Kadonaga[d], Michael G. Rosenfeld[g] and Christopher K. Glass[a,e,*]

[a]Department of Cellular and Molecular Medicine, University of California San Diego, La Jolla, CA, USA
[b]Stem Cell Program, University of California San Diego, La Jolla, CA, USA
[c]Institute for Genomic Medicine, University of California San Diego, La Jolla, CA, USA
[d]Department of Molecular Biology, University of California San Diego, La Jolla, CA, USA
[e]Department of Medicine, University of California San Diego, La Jolla, CA, USA
[f]Division of Allergy and Immunology, Cincinnati Children's Hospital Medical Center, Cincinnati, OH, USA
[g]Department and School of Medicine, University of California San Diego, La Jolla, CA, USA
[h]Gene Expression Laboratory, Salk Institute for Biological Studies, La Jolla, CA, USA
[i]Faculty of Biology, Department II, Ludwig-Maximilians Universität München, Planegg-Martinsried, Germany
[j]Department of Bioengineering, Jacobs School of Engineering, University of California San Diego, La Jolla, CA, USA
[k]Division of Metabolic Medicine, Research Center for Advanced Science and Technology, The University of Tokyo, Tokyo, Japan
[l]Division of Molecular Physiology and Metabolism, Tohoku University Graduate School of Medicine, Sendai, Japan

Corresponding author: Christopher K. Glass
Email: ckg@ucsd.edu

This PDF file includes:

Supporting text
Figures S1 to S9
Table S1

Supporting text

Materials and Methods

Animal studies

All animal procedures were in accordance with University of California San Diego research guidelines for the care and use of laboratory animals. Mice were maintained under a 12 hr light/12 hr dark cycle at constant temperature (20-23°C) with free access to food and water. Animals were fed a normal chow diet (T8604, Envigo). *Ncor*$^{f/f}$ and *Ncor*$^{f/f}$ *LysM-Cre* mice were described previously (1). *Pgc1b*$^{f/f}$ mice were described previously (2). *Ncor*$^{f/f}$ and *Pgc1b*$^{f/f}$ mice were used as WT mice for *Ncor*$^{f/f}$ *LysM-Cre* and *Pgc1b*$^{f/f}$ *LysM-Cre* mice, respectively.

In Vivo LPS shock study

8- to 12-week-old C57BL/6 male mice were intraperitoneally injected with 6 mg/kg of LPS (Sigma-Aldrich) and observed continuously every 4 hrs. Intraperitoneal injection of 10 mg/kg RGFP966 (Selleckchem) or 10% DMSO as vehicle control was performed 6 hrs before LPS injection. Identities were blinded from the experimenter until the end of studies. All surviving animals were humanely euthanized at 96 hrs after LPS injection.

Antibodies

Mouse monoclonal antibody immunoglobulin G-Y8129 (IgG-Y8129) against mouse NCoR (amino acids 1817-1879) was produced by immunizing mice with gp64 fusion protein expressed by baculoviral system as described previously (3). A list of other antibodies used in this article is shown in Table S1.

Bone marrow-derived osteoclast culture

Bone marrow cells were obtained by flushing the tibia and femur from 8- to 12-week-old C57BL/6 mice with alpha MEM (Sigma-Aldrich) containing 10% FBS, 1% penicillin/streptomycin+L-glutamine and lysed using red blood cell lysis buffer. 48 million cells per 15 cm tissue culture plates for ChIP-seq were cultured in alpha MEM containing 10% FBS, 1% penicillin/streptomycin+L-glutamine and 10 ng/ml M-CSF for 3 days to generate osteoclast precursor cells. After non-adherent cells were washed off with alpha MEM, adherent osteoclast precursor cells were cultured in alpha MEM containing 10% FBS, 1% penicillin/streptomycin+L-glutamine, 10 ng/ml M-CSF and 50 ng/ml RANKL for 4 days to differentiate to osteoclasts. The M-CSF plus RANKL containing culture media was replaced every 2 days.

Immunoblotting and immunoprecipitation

Whole-cell lysates were prepared as previously described (4) with modifications as follows. Cells were sonicated in cell lysis buffer (50 mM HEPES-KOH (pH 7.9), 150 mM NaCl, 1.5 mM $MgCl_2$, 1% NP-40, 1 mM PMSF (Sigma-Aldrich), 1X protease inhibitor cocktail (Sigma-Aldrich)) by ultrasound homogenizer (Bioruptor, Diagenode) for 10 min at 4°C. For immunoblotting, aliquots of whole-cell lysate were boiled at 95°C for 5 min in NuPAGE™ LDS Sample Buffer (Thermo Fisher Scientific) with NuPAGE™ Sample Reducing Agent (Thermo Fisher Scientific), subjected to SDS-PAGE, and transferred to immobilon-P transfer membranes (Merck Millipore). Immunodetection was carried out with the indicated antibodies (Table S1) and bound antibodies were visualized with peroxidase-conjugated affinity-purified donkey anti-mouse or anti-rabbit IgG (Dako), or mouse monoclonal anti-rabbit IgG light chain (Abcam) using SuperSignal™ West Femto Maximum Sensitivity Substrate (Thermo Fisher Scientific) or Luminate™ Forte Western HRP Substrate (Merck Millipore), and luminescence images were analyzed by ChemiDoc XRS+ System (Bio-Rad Laboratories). Uncropped images of blots are shown in Fig. S7, S8 and S9. For immunoprecipitation, whole-cell lysates were immunoprecipitated in cell lysis buffer (50 mM HEPES-KOH (pH 7.9), 150 mM NaCl, 1.5 mM $MgCl_2$, 1% NP-40, 1mM PMSF, 1X protease inhibitor cocktail) by wheel rotating overnight at 4°C in the presence of Dynabeads protein G (Thermo Fisher Scientific) and each antibody described in Table S1. After immunoprecipitation, beads were washed three times with cell lysis buffer and three times with PBS, and then eluted with sample buffer. The elution was subjected to immunoblotting as described above.

HDAC3 activity assay

HDAC3 activity in BMDMs was measured using a HDAC3 Activity Assay Kit (Sigma-Aldrich) according to the manufacturer's protocol. The deacetylase activity of immunoprecipitated HDAC3 or PGC1β with anti-HDAC3 or PGC1β antibody in 300 μg of whole-cell lysate as described in **Immunoblotting and immunoprecipitation**. Briefly, HDAC3 assay buffer in the presence or absence of 10 μM GSK8612 was added to the HDAC3 or PGC1β antibody-Dynabeads Protein G complex and incubated for 10 min at 37°C. Then HDAC3 assay buffer containing HDAC3 substrate with a fluorophore [R-H-K-K(Ac)-AFC] were mixed with the complex and incubated for 30 min at 37°C. Once the incubation was complete, developing solution was added and incubated for 5 min at 37°C. The reaction solutions were transferred to a 96-well plate and the fluorescent signals were determined at 380 nm of excitation and 500 nm of emission.

Histone acetyltransferase (HAT) activity assay

HAT activity in BMDMs was measured using a HAT Assay Kit (Active Motif) according to the manufacturer's protocol. The HAT activity of immunoprecipitated NCoR or PGC1β with anti-NCoR or PGC1β antibody in 300 μg of whole-cell lysate as described in **Immunoblotting and immunoprecipitation**. Briefly, 50 μl of 1X assay buffer containing 50 μM acetyl-CoA and 50 μM Histone H3 was added to the NCoR or PGC1β antibody-Dynabeads Protein G complex and incubated for 30 min at room temperature. Once the incubation was complete, 50 μl of stop solution was mixed. Then 100 μl of developing solution was added and incubated for 15 min in the dark at room temperature. The reaction solutions were transferred to a 96-well plate and the fluorescent signals were determined at 380 nm of excitation and 450 nm of emission.

ATAC-seq library preparation

70000 cultured cells were washed once with PBS and once with cold lysis buffer (10 mM Tris-HCl (pH 7.4), 10 mM NaCl, 3 mM $MgCl_2$, 0.1% NP-40). The cells were suspended in 50 μl of 1X Reaction Buffer (25 μl of Tagment DNA Buffer, 2.5 μl of Tagment DNA enzyme I, and 22.5 μl of water) (Nextera DNA Library Preparation Kit, Illumina) as previously described (5). Transposase reactions were carried out at 37°C for 30 min, and then DNA was purified using ChIP DNA Clean & Concentrator Kit (Zymo Research). DNA was amplified using the Nextera primer Ad1 and a unique Ad2.n barcoding primer using NEBNext High-Fidelity 2X PCR Master Mix (NEB) for 7 cycles. The amplified libraries were purified with 2 μl of SpeedBeads (GE Healthcare) in 20% PEG 8000/2.5 M NaCl (Final 13% PEG 8000), eluted with 15 μl of EB (Zymo Research), size selected using PAGE/TBE gel (Invitrogen) for 175-225 bp fragments by gel extraction, and single-end sequenced on HiSeq 4000 (Illumina).

ChIP-seq library preparation

Chromatin immunoprecipitation (ChIP) was performed in biological replicates as previously described (6) with modifications as follows. For H3K27ac ChIP assays, cells were cross-linked with 1% (vol/vol) formaldehyde (Thermo Fisher Scientific) in PBS for 10 min at room temperature. For NCoR, HDAC3, p65, Fosl2, PU.1, PGC1β, ERK1 and p300 ChIP assays, cells were cross-linked with 2 mM disuccinimidyl glutarate (DSG) (ProteoChem) in PBS for 30 min at room temperature, and then directly a second cross-linking was performed by the addition of 1% (vol/vol) formaldehyde in PBS for 10 min. The cross-linking reaction was quenched by 0.125 M glycine (Sigma-Aldrich). Cells were washed once with ice-cold PBS and pelleted at 1000 g for 5 min at 4°C. Cross-linked cells were resuspended in ice-cold hypotonic buffer (10 mM HEPES-KOH (pH 7.9), 85 mM KCl, 1 mM EDTA, 1% NP-40, 1 mM PMSF, 0.5 mM sodium butyrate (Sigma-Aldrich), 1X protease inhibitor cocktail), and centrifuged at 1000 g for 5 min at 4°C to obtain a nuclear fraction. Nuclear pellets were resuspended in 100 μl of either LB3 buffer (10 mM Tris-HCl (pH 7.5), 100 mM NaCl, 1 mM EDTA, 0.5 mM EGTA, 0.1% sodium deoxycholate, 0.5% N-laurosylsarcosine, 1 mM PMSF, 0.5 mM sodium butyrate, 1X protease inhibitor cocktail, for H3K27ac ChIP) or PIPA-NR buffer (20 mM Tris-HCl (pH 7.5), 150 mM NaCl, 1 mM EDTA, 0.5 mM EGTA, 0.4% sodium deoxycholate, 0.1% SDS, 1% NP-40, 0.5 mM DTT (Thermo Fisher Scientific), 1 mM PMSF, 0.5 mM sodium butyrate, 1X protease inhibitor cocktail, for NCoR, HDAC3, p65, Fosl2, PU.1, PGC1β, ERK1 and p300 ChIP). Chromatin DNA was sonicated in a 96 Place microTUBE Rack (Covaris)

using a Covaris E220 for 12-18 cycles with the following setting: time, 60 seconds; duty, 5.0; PIP, 140; cycles, 200; amplitude, 0.0; velocity, 0.0; dwell, 0.0. Samples were centrifuged at 15000 rpm for 10 min at 4°C, and the supernatant was used for immunoprecipitation. LB3 lysate was diluted 1.1-fold with 10% Triton X-100. 1% of the lysate was kept as ChIP input. The lysates were rotated with each antibody pre-bound to 20 μl of beads (10 μl of Dynabeads protein A (Thermo Fisher Scientific) + 10 μl of Dynabeads protein G) overnight at 4°C. After the immunoprecipitation, beads were collected on a magnet and washed three times each with wash buffer I (20 mM Tris-HCl (pH 7.5), 150 mM NaCl, 1% Triton X-100, 0.1% SDS, 2 mM EDTA, 1 mM PMSF, 0.5 mM sodium butyrate, 1X protease inhibitor cocktail), wash buffer III (10 mM Tris-HCl (pH 7.5), 250 mM LiCl, 1% Triton X-100, 0.7% sodium deoxycholate, 1 mM EDTA, 1 mM PMSF, 0.5 mM sodium butyrate, 1X protease inhibitor cocktail) and twice with TET (10 mM Tris-HCl (pH 7.5), 1 mM EDTA, 0.2% Tween-20, 1 mM PMSF, 0.5 mM sodium butyrate, 1X protease inhibitor cocktail), and then resuspended in 25 μl of TT (10mM Tris-HCl (pH 7.5), 0.05% Tween-20). The immunoprecipitated chromatin samples were used for library preparation with NEBNext Ultra II Library kit (NEB) according to the manufacturer's instructions. DNA in 46.5 μl of NEB reactions was crosslinks reversed by adding 4 μl of 10% SDS, 4.5 μl of 5 M NaCl, 3 μl of 500 mM EDTA, 1 μl of 20 mg/ml proteinase K (NEB) and 20 μl of water by incubation for 1 hr at 55°C, and then 30 min at 65°C. DNA was purified with 2 μl of SpeedBeads in 20% PEG 8000/1.5 M NaCl (Final 12% PEG 8000), and eluted with 25 μl of TT. The eluted DNA was amplified for 14 cycles in 50 μl of PCR reactions using NEBNext High-Fidelity 2X PCR Master Mix and 0.5 mM each of primers Solexa 1GA and Solexa 1GB. The amplified libraries were purified with 2 μl of SpeedBeads in 20% PEG 8000/2.5 M NaCl (Final 13% PEG 8000), eluted with 20 μl of TT, size selected using PAGE/TBE gel for 225-325 bp fragments by gel extraction, and single-end sequenced on HiSeq 4000. ChIP input material (1% of sheared DNA) in 46.5 μl of TE was crosslinks reversed by adding 4 μl of 10% SDS, 4.5 μl of 5 M NaCl, 3 μl of 500 mM EDTA, 1 μl of 20 mg/ml proteinase K and 20 μl of water by incubation for 1 hr at 55°C, and then 30 min at 65°C. The input DNA was purified with SpeedBeads as described above, and eluted with 25 μl of TT. The eluted input DNA was prepared for libraries and amplified as described for ChIP samples.

RNA-seq library preparation

Total RNA was isolated from culture cells and purified using a Direct-zol RNA MicroPrep Kit (Zymo Research) as described by the manufacturer. mRNAs were enriched by incubation with Oligo $d(T)_{25}$ Magnetic Beads (NEB). To fragment Poly A-enriched mRNA, mRNAs were incubated in 2X Superscript III first-strand buffer (Thermo Fisher Scientific) with 10 mM DTT at 94°C for 9 min. The 10 μl of fragmented mRNAs were incubated with 0.5 μl of Random primers (3 μg/μl) (Thermo Fisher Scientific), 0.5 μl of Oligo dT primer (50 μM) (Thermo Fisher Scientific), 0.5 μl of SUPERase-In (Ambion) and 1 μl of dNTPs (10 mM) (Thermo Fisher Scientific) at 50°C for 1 min. After the incubation, 5.8 μl of water, 1 μl of DTT (100 mM), 0.1 μl of Actinomycin D (2 μg/μl) (Sigma-Aldrich), 0.2 μl of 1% Tween-20 and 0.5 μl of Superscript III (Thermo Fisher Scientific) were added and incubated on the following conditions: 25°C for 10 min, 50°C for 50 min, and 4°C hold. The mixture was purified with RNAClean XP beads (Beckman Coulter) as described by the manufacturer and eluted with 10 μl of water. For second-strand synthesis with dUTP, the RNA/cDNA double-stranded hybrid was then added to 1.5 μl of Blue Buffer (Enzymatics), 1.1 μl of dUTP mix (10 mM dATP, dCTP, dGTP and 20 mM dUTP) (Promega), 0.2 μl of RNase H (5 U/μl) (Enzymatics), 1.05 μl of water, 1 μl of DNA polymerase I (Enzymatics) and 0.15 μl of 1% Tween-20. The mixture was incubated at 16°C for 2.5 hrs. The resulting dUTP-marked dsDNA was purified with 2 μl of SpeedBeads in 20% PEG 8000/2.5M NaCl (final 13% PEG 8000), and eluted with 40 μl of EB. The eluted dsDNA was carried out end repair by blunting, A-tailing and adapter ligation as previously described (7) using barcoded adapters (Bioo Scientific). The end repaired dsDNA was amplified for 14-16 cycles using 0.05 U/μl KAPA High Fidelity HotStart DNA polymerase (Kapa Biosystems) and 1 μM each of primers Solexa 1GA and Solexa 1GB. The amplified libraries were selected using PAGE/TBE gel for 225-325 bp fragments by gel extraction, and single-end sequenced on HiSeq 4000.

RNA-seq analysis
FASTQ files were processed to assess quality by determining general sequencing bias, clonality and adapter sequence contamination. RNA sequencing reads were aligned to the mm10 mouse reference genome using STAR (8). Gene expression levels were calculated using HOMER (7) by counting all strand specific reads within exons. Only the most abundant transcripts, including multiple alternative variants, were selected for each gene, and the genes with a length smaller than 250 bp were removed. Transcripts per million (TPM) were used to evaluate the correlation among replicates. Differential gene expression was calculated using DESeq2 (9) to assess both biological and technical variability between experiments. Unsupervised hierarchical clustering was used to cluster the gene expression in the heatmaps.

ATAC-seq and ChIP-seq analysis
FASTQ files were mapped to the mm10 mouse reference genome with Bowtie2 (10). Peaks were called with HOMER (findPeaks) using parameters "-style factor -minDist 200 -size 200". After merging these peaks, correlations among replicates from the same cell subset/treatment were evaluated by correlation using tag counts. The two most highly correlated samples were used for identifying the most robust peaks using the irreproducible discovery rate (IDR) method (11). For this step, peaks were called with HOMER's findPeaks, using parameters "-L 0 -C 0 -fdr 0.9 -minDist 200 -size 200". IDR peaks from different conditions involved in a comparison merged with HOMER's mergePeaks and annotated with HOMER's annotatePeaks.pl. The raw tags of all samples which had reasonable correlation were quantified with HOMER (annotatePeaks.pl) using parameter "-noadj". Peaks which contained at least 4 tags in at least 1 sample were used to identify differentially bounded peaks (DBP) by DESeq2. Peaks were categorized as distal peaks which are 2 kb away from known TSS and promotor peaks which are located within 2 kb region of known TSS sites. Histone marks, such as H3K27ac single, were quantified by either ATAC IDR peaks under the same conditions or transcription factor ChIP IDR peaks. ATAC peak quantification was normalized to the total tags in peaks, while the ChIP peak single was normalized to the sequence depth.

Motif analysis
To identify motifs enriched in peak regions over the background, HOMER's motif analysis "findMotifsGenome.pl" including known default motifs and *de novo* motifs was used (7). The background peaks used either from random genome sequences or from peaks in comparing condition were indicated throughout the main text and in the figure legends.

Data visualization
ChIP-seq data were visualized in the UCSC genome browser (12).

Statistical analysis
The significance of differences in the experimental data were determined using GraphPad Prism 8.0 software. All data involving statistics are presented as mean ± s.d. The number of replicates and the statistical test used are described in the figure legends.

Figures

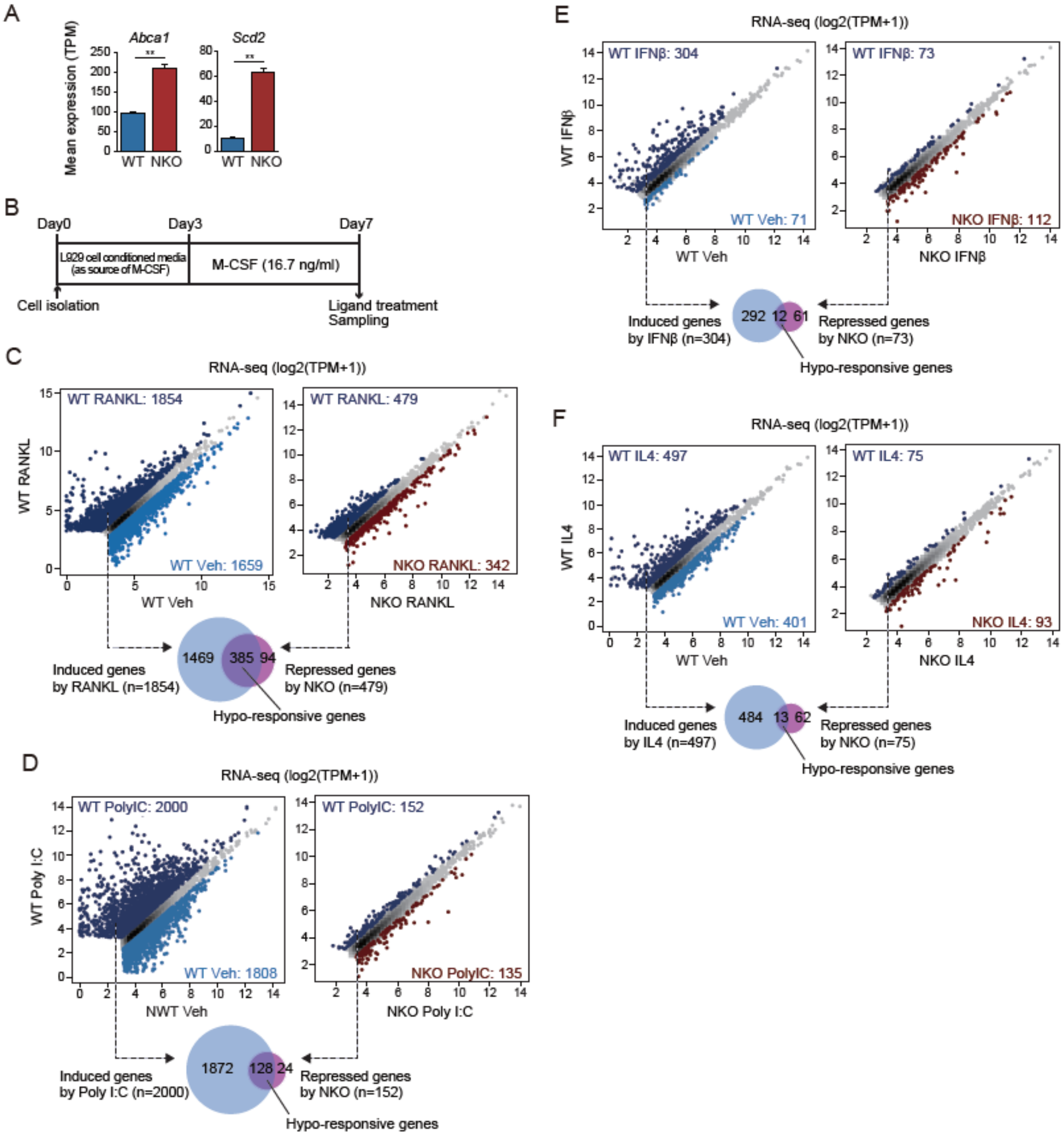

Fig. S1
(A) Bar plots for expression of *Abca1* and *Scd2* in BMDMs from WT and NKO mice. The significance symbols indicate statistical significance, **p-adj < 0.01 reported by DESeq2 using the Benjamini-Hochberg method for the multiple-testing correction.
(B) BMDMs at Day7 after differentiation were treated with or without each ligand, and then subjected to experiments.
(C, D, E, F) Scatter plots of RNA-seq data showing RANKL (C), Poly I:C (D), IFNβ (E) or IL4 (F)-regulated gene expression and NKO-regulated gene expression in the presence of RANKL, Poly I:C, IFNβ or IL4 (light blue dots in left panels: significantly RANKL, Poly I:C, IFNβ or IL4-suppressed genes, dark blue dots in left panels: significantly RANKL, Poly I:C, IFNβ or IL4-induced genes, dark red dots in right panels: significantly NKO-induced genes, dark blue dots in right panels: significantly NKO-suppressed genes, FDR < 0.05, FC > 1.5).

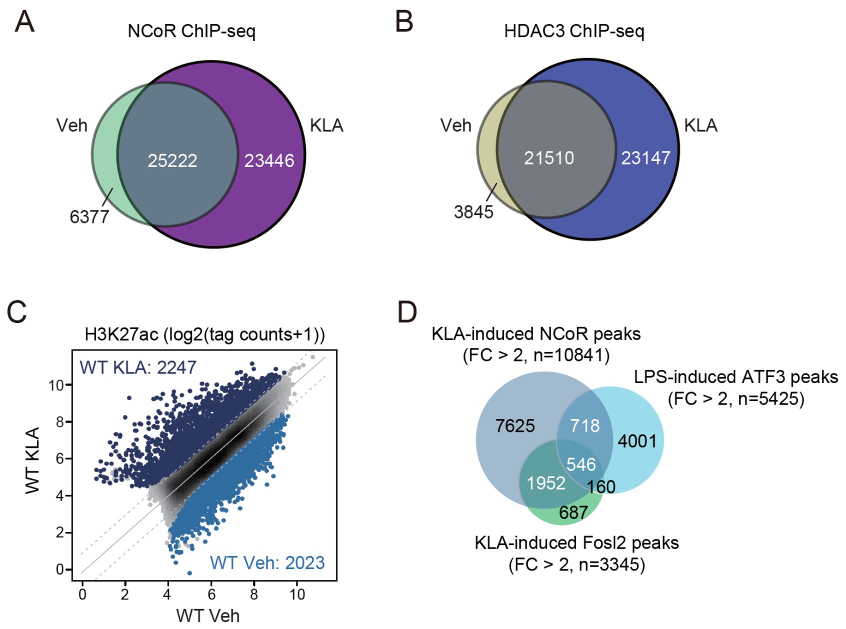

Fig. S2
(A, B) The overlap between IDR-defined NCoR (A) or HDAC3 (B) ChIP-seq peaks at Veh and KLA is shown by Venn diagram.
(C) Scatter plot of normalized H3K27ac ChIP-seq tags having at least 16 tags associated with ATAC-seq IDR peaks at Veh in a 1000 bp window. KLA-induced H3K27ac peaks (FDR < 0.05, FC > 2) are color-coded (light blue dots: significantly lost H3K27ac by KLA treatment, dark blue dots: significantly gained H3K27ac by KLA treatment).
(D) The overlaps between KLA- or LPS-induced NCoR, Fosl2 and ATF3 (13) ChIP-seq peaks (FC > 2) are shown by Venn diagram.

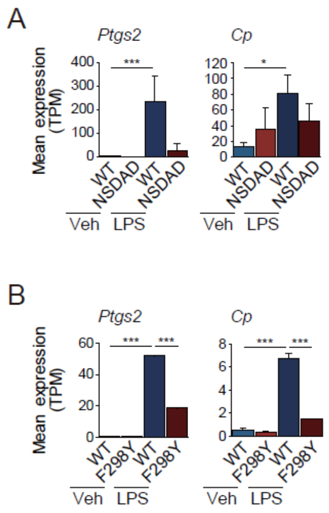

Fig. S3

(A, B) Bar plots for expression of *Ptgs2* and *Cp* in peritoneal macrophages from LPS-injected WT and NSDAD mice (A) and HDAC3 WT or Y298F-rescued HDAC3-deficient BMDMs treated with or without LPS for 4 hours (B) (13). The significance symbols indicate statistical significance, *p-adj < 0.05, ***p-adj < 0.001 reported by DESeq2 using the Benjamini-Hochberg method for the multiple-testing correction.

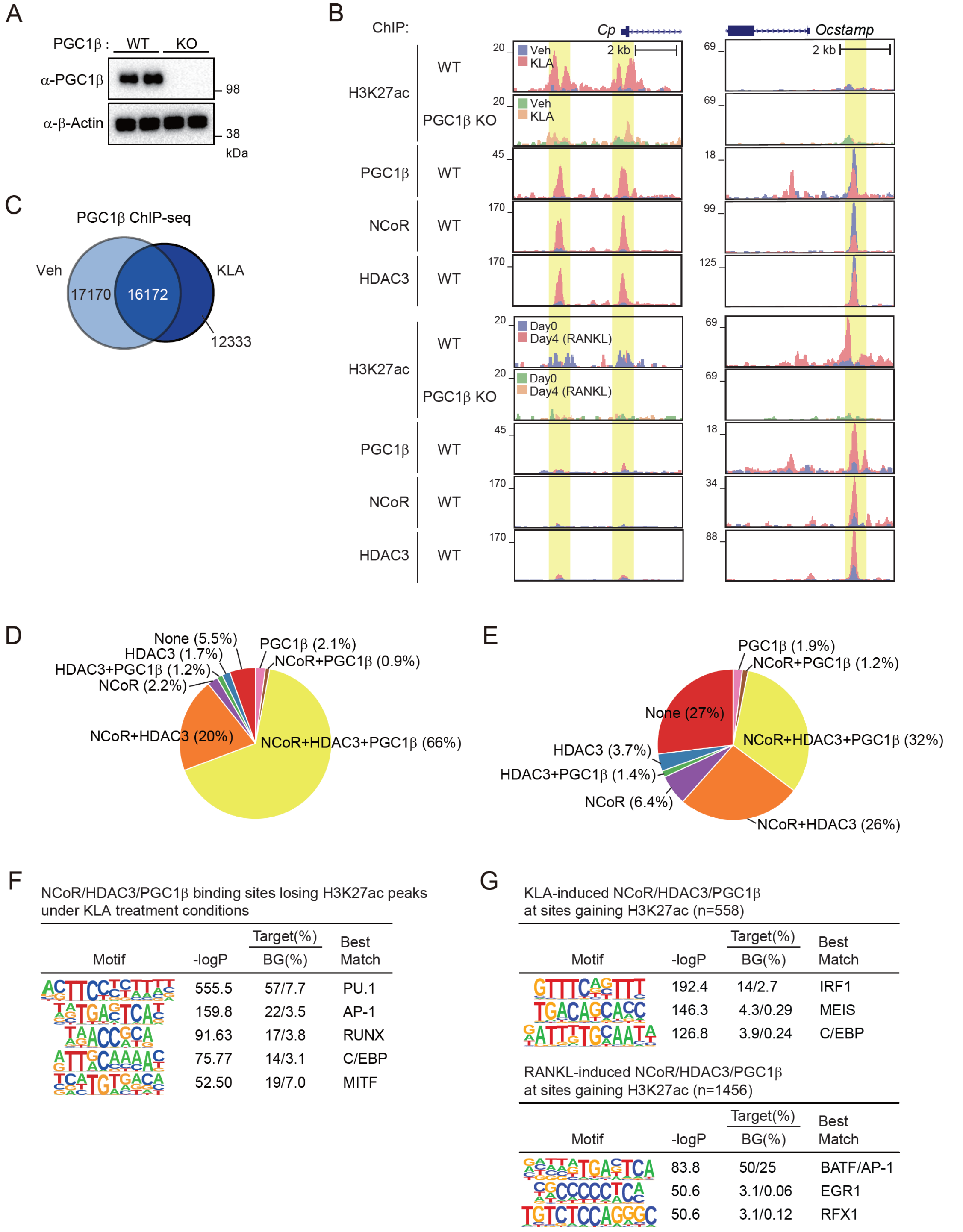

Fig. S4

(A) Immunoblot analysis for PGC1β protein in BMDMs from *Pgc1b$^{f/f}$* (WT) and *Pgc1b$^{f/f}$ LysM-Cre* (KO) mice (12-week-old male, n=2 each). Uncropped images of the blots are shown in Fig. S9. (B) Genome browser tracks of H3K27ac, PGC1β, NCoR and HDAC3 ChIP-seq peaks in the vicinity of the *Cp* and *Ocstamp* loci. Yellow shading: lost H3K27ac by PGC1β KO at KLA- (top panels) or RANKL- (bottom panels) induced NCoR, HDAC3 and PGC1β binding regions.

(C) The overlap between IDR-defined PGC1β ChIP-seq peaks at Veh and KLA is shown by Venn diagram.
(D) The overlaps of ATAC-defined gained H3K27ac peaks in the presence of KLA (n=2247 in Fig. S2*C*) with NCoR, HDAC3 and/or PGC1β ChIP-seq peaks are shown by pie chart.
(E) The overlaps of ATAC-defined lost H3K27ac peaks in the presence of KLA (n=2023 in Fig. S2*C*) with NCoR, HDAC3 and/or PGC1β ChIP-seq peaks are shown by pie chart.
(F) *De novo* motif enrichment analysis of NCoR/HDAC3/PGC1β binding sites losing H3K27ac peaks under KLA treatment conditions (n=648 in Fig. S4*E*) using a GC-matched genomic background.
(G) *De novo* motif enrichment analysis of KLA- or RANKL-induced NCoR/HDAC3/PGC1β at sites gaining H3K27ac peaks (Fig. 4*E*) using RANKL- or KLA-induced NCoR/HDAC3/PGC1β at sites gaining H3K27ac peaks as a background, respectively.

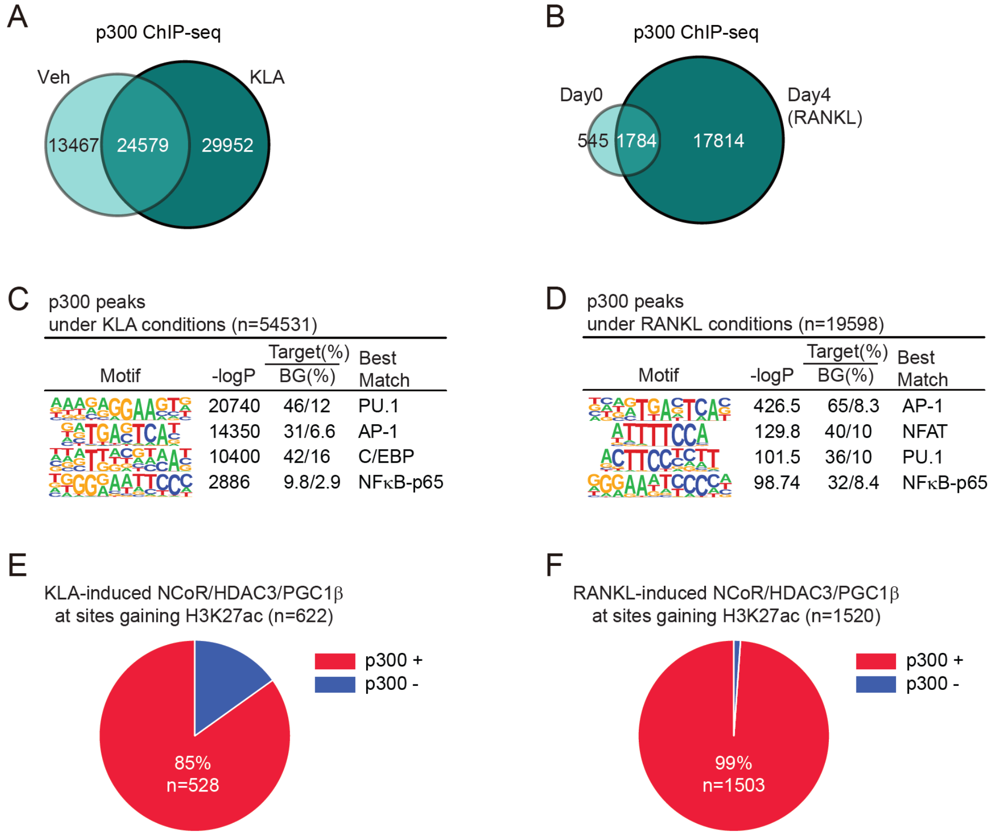

Fig. S5
(A, B) The overlap between IDR-defined p300 ChIP-seq peaks at Veh and KLA (A) or RANKL (B) is shown by Venn diagram.
(C, D) *De novo* motif enrichment analysis of p300 ChIP-seq peaks in the presence of KLA (n=54531 in Fig. S5*A*) (C) or RANKL (n=19598, Fig. S5*B*) (D) using a GC-matched genomic background.
(E, F) The colocalization of p300 ChIP-seq peaks with KLA- (E) or RANKL- (F) (14) induced NCoR/HDAC3/PGC1β-associated gained H3K27ac peaks is shown by pie chart.

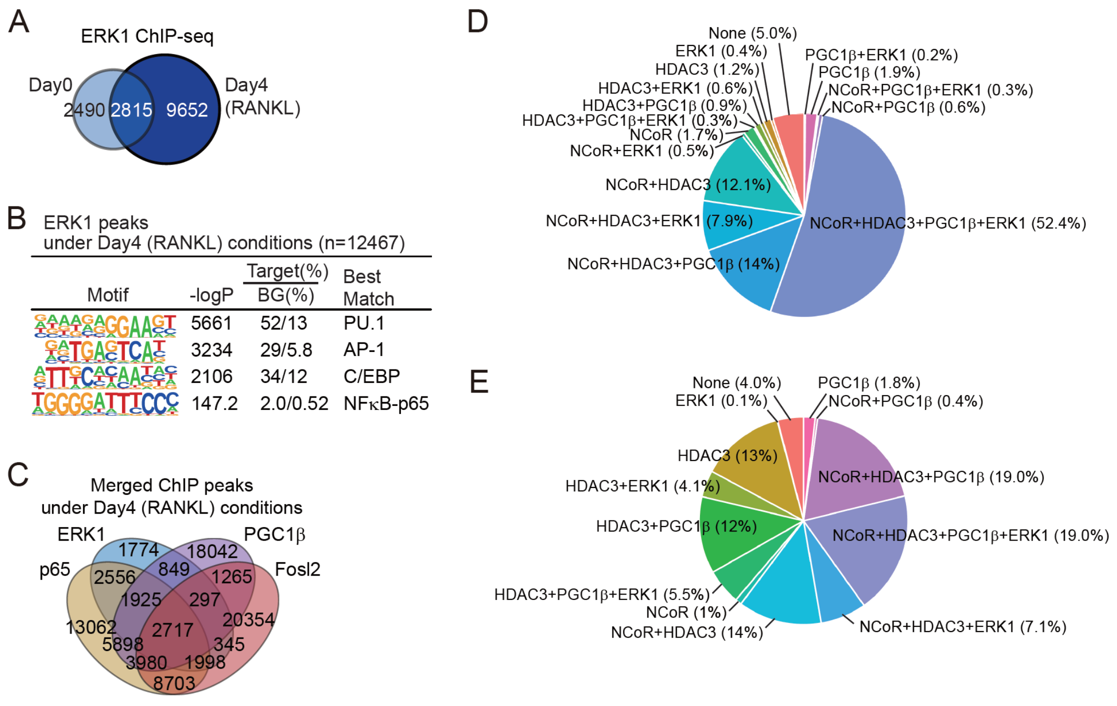

Fig. S6
(A) The overlap between IDR-defined ERK1 ChIP-seq peaks at Day0 and Day4 after RANKL treatment is shown by Venn diagram.
(B) *De novo* motif enrichment analysis of ERK1 peaks under Day4 after RANKL treatment (n=12467 in Fig. S6*A*) using a GC-matched genomic background.
(C) The overlap between IDR-defined ERK1, PGC1β, p65 and Fosl2 ChIP-seq peaks under Day4 after RANKL treatment (14) is shown by Venn diagram.
(D) The overlaps of ATAC-defined gained H3K27ac peaks in the presence of KLA (n=2247 in Fig. S2*C*) with NCoR, HDAC3, PGC1β and/or ERK1 ChIP-seq peaks are shown by pie chart.
(E) The overlaps of ATAC-defined gained H3K27ac peaks in the presence of RANKL (n=1525 in Figure S2E from (14)) with NCoR, HDAC3, PGC1β and/or ERK1 ChIP-seq peaks are shown by pie chart.

Fig. 4*A*

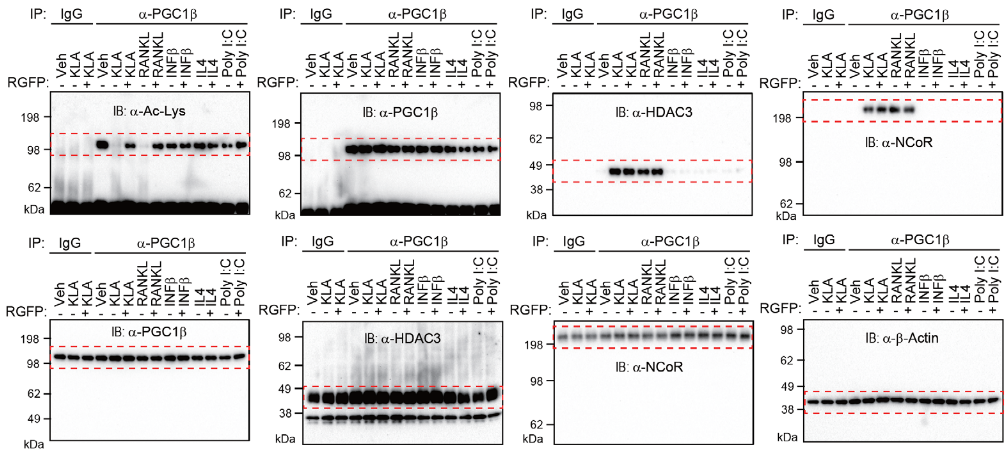

Fig. 5*C*

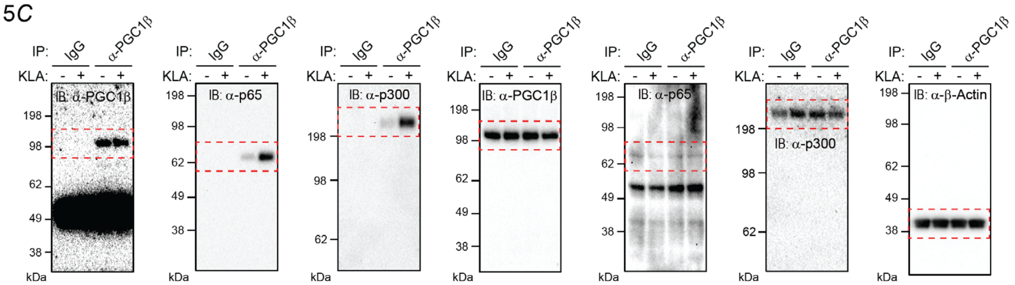

Fig. 5*E*

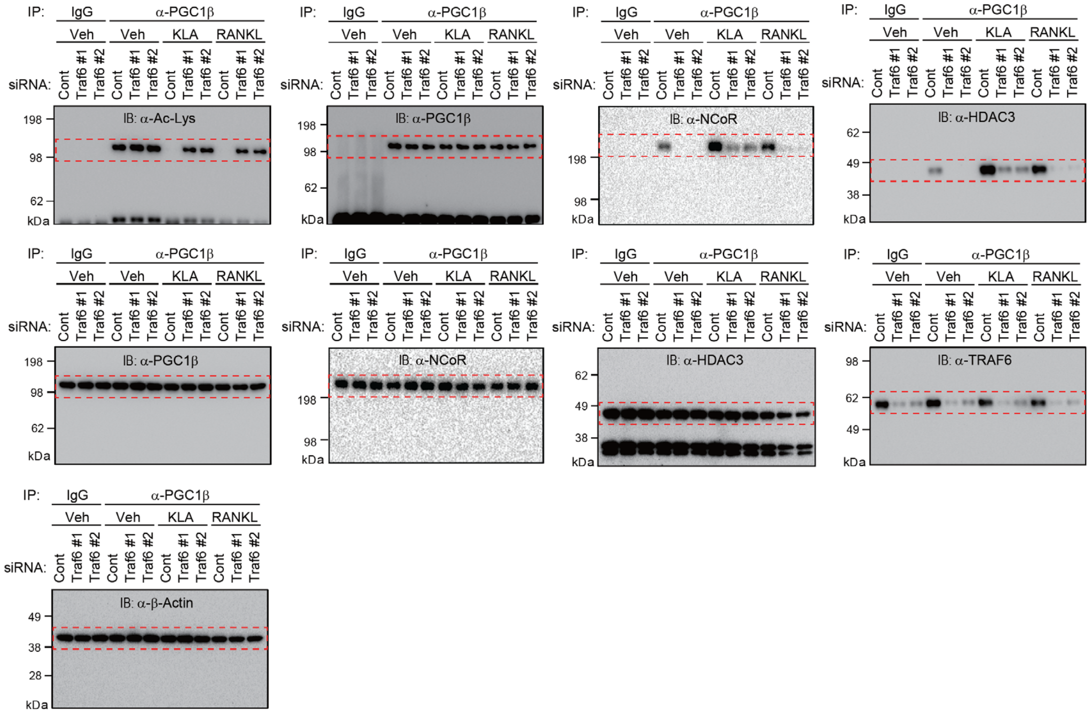

Fig. S7
Representative original images of immunoblot analysis for Fig. 4*A*, 5*C* and 5*E*

Fig. 5*G*

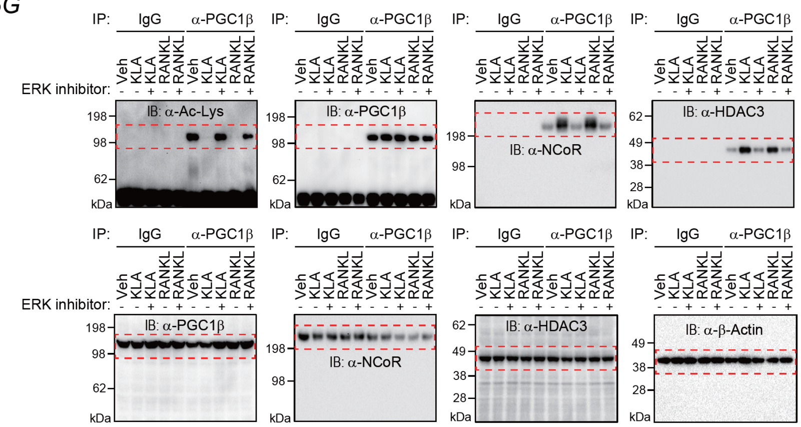

Fig. 5*H*

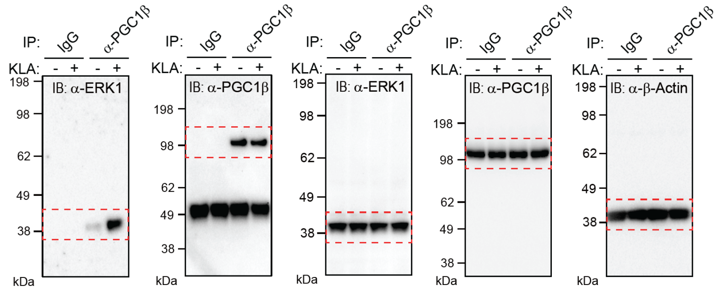

Fig. 5*I*

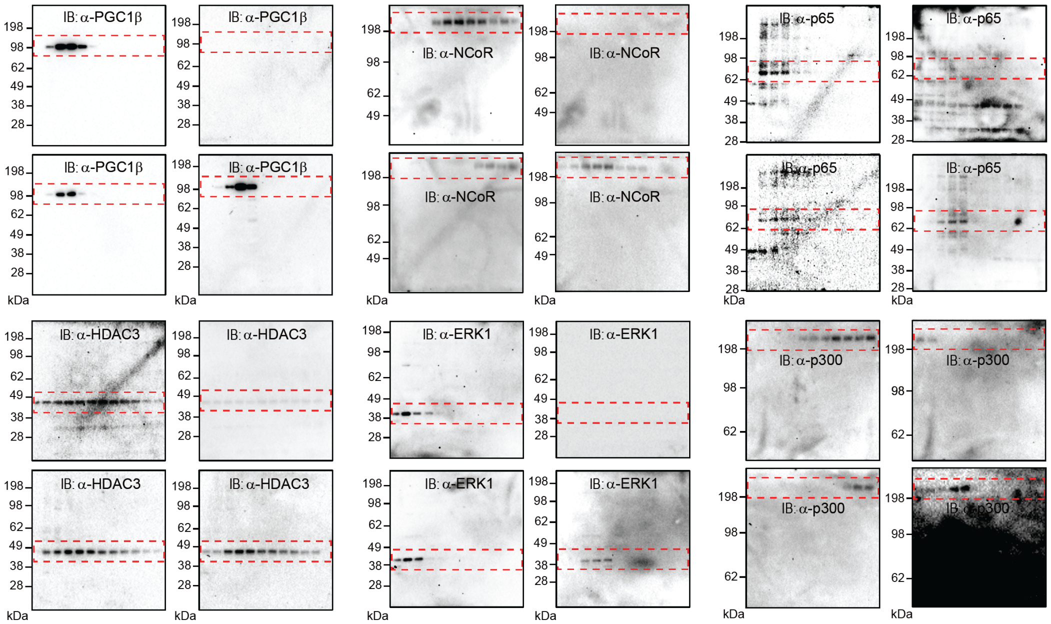

Fig. S8
Representative original images of immunoblot analysis for Fig. 5*G*, 5*H* and 5*I*

Fig. 7*B*

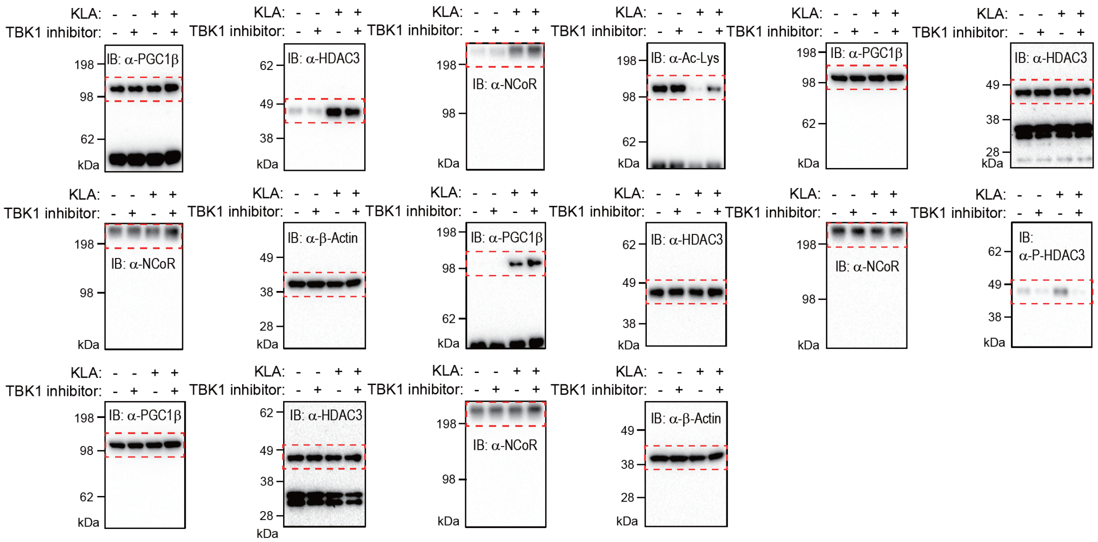

Fig. S4*A*

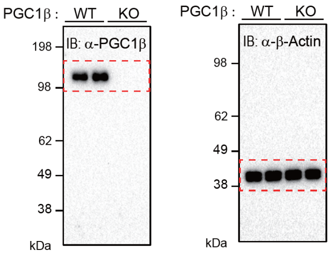

Fig. S9
Representative original images of immunoblot analysis for Fig. 7*B* and S4*A*

Table S1
Detailed information about antibodies

Antibody		Source	Catalog No / Clone No	Dilutions / Concentrations
anti-NCoR	Monoclonal	Dr. Hamakubo	IgG-Y8129	5 μg/assay for ChIP
				2 μg/assay for IP
				1 μg/ml for IB
anti-HDAC3	Polyclonal	GeneTex	GTX113303	3 μg/assay for ChIP
				2 μg/assay for IP
				0.32 μg/ml for IB
anti-PU.1	Polyclonal	Santa Cruz	sc-352 / T-21	2 μg/assay for ChIP
anti-p65	Polyclonal	Santa Cruz	sc-372 / C-20	2 μg/assay for ChIP
anti-p65	Monoclonal	Santa Cruz	sc-8008X / F-6	2 μg/ml for IB
anti-Fosl2	Monoclonal	Santa Cruz	sc-166102 / G-5	4 μg/assay for ChIP
anti-PGC1β	Monoclonal	Abcam	ab176328	0.1 μg/assay for ChIP
				0.1 μg/assay for IP
				0.093 μg/ml for IB
anti-ERK1	Monoclonal	Abcam	ab119357	2 μg/assay for ChIP<br>1:1000 for IB
Anti-p300	Monoclonal<br>Monoclonal	Diagenode<br>Millipore	C15200211<br>05-257	2 μg each/assay for ChIP
anti-p300	Monoclonal	Cell signaling Technology	#70088	1:1000 for IB
anti-Acetylated-Lysine	Monoclonal	Cell signaling Technology	#9441	1:1000 for IB
anti-H3K27ac	Polyclonal	Active Motif	#39133	2 μg/assay for ChIP
anti-TRAF6	Monoclonal	Santa Cruz	sc-8409 / D-10	0.2 μg/assay for IB
anti-Phospho-HDAC3 (S424)	Polyclonal	Cell Signaling Technology	#3815	1:1000 for IB
anti-β-Actin	Monoclonal	Sigma-Aldrich	A2228 / AC-74	1:5000 for IB

SI References

1. P. Li *et al.*, NCoR repression of LXRs restricts macrophage biosynthesis of insulin-sensitizing omega 3 fatty acids. *Cell* **155**, 200-214 (2013).
2. J. Sonoda, I. R. Mehl, L. W. Chong, R. R. Nofsinger, R. M. Evans, PGC-1beta controls mitochondrial metabolism to modulate circadian activity, adaptive thermogenesis, and hepatic steatosis. *Proc Natl Acad Sci U S A* **104**, 5223-5228 (2007).
3. T. Tanaka *et al.*, The generation of monoclonal antibodies against human peroxisome proliferator-activated receptors (PPARs). *J Atheroscler Thromb* **9**, 233-242 (2002).
4. Y. Abe *et al.*, Histone demethylase JMJD1A coordinates acute and chronic adaptation to cold stress via thermogenic phospho-switch. *Nat Commun* **9**, 1566 (2018).
5. J. D. Buenrostro, P. G. Giresi, L. C. Zaba, H. Y. Chang, W. J. Greenleaf, Transposition of native chromatin for fast and sensitive epigenomic profiling of open chromatin, DNA-binding proteins and nucleosome position. *Nat Methods* **10**, 1213-1218 (2013).
6. S. Heinz *et al.*, Transcription Elongation Can Affect Genome 3D Structure. *Cell* **174**, 1522-1536.e1522 (2018).
7. S. Heinz *et al.*, Simple combinations of lineage-determining transcription factors prime cis-regulatory elements required for macrophage and B cell identities. *Mol Cell* **38**, 576-589 (2010).
8. A. Dobin *et al.*, STAR: ultrafast universal RNA-seq aligner. *Bioinformatics* **29**, 15-21 (2013).
9. M. I. Love, W. Huber, S. Anders, Moderated estimation of fold change and dispersion for RNA-seq data with DESeq2. *Genome Biol* **15**, 550 (2014).
10. B. Langmead, S. L. Salzberg, Fast gapped-read alignment with Bowtie 2. *Nat Methods* **9**, 357-359 (2012).
11. Q. Li, J. B. Brown, H. Huang, P. J. Bickel, Measuring reproducibility of high-throughput experiments. *The Annals of Applied Statistics* **5**, 1752-1779, 1728 (2011).
12. W. J. Kent *et al.*, The human genome browser at UCSC. *Genome Res* **12**, 996-1006 (2002).
13. H. C. B. Nguyen, M. Adlanmerini, A. K. Hauck, M. A. Lazar, Dichotomous engagement of HDAC3 activity governs inflammatory responses. *Nature* **584**, 286-290 (2020).
14. Y. Abe *et al.*, RANK ligand converts the NCoR/HDAC3 co-repressor to a PGC1β- and RNA-dependent co-activator of osteoclast gene expression. *Mol Cell* **83**, 3421-3437.e3411 (2023).
